# Multichamber Myocardial Strain for Cardiovascular Risk Stratification in Liver Transplant Recipients Using Interpretable Machine Learning

**DOI:** 10.1016/j.jacadv.2026.103170

**Published:** 2026-08-26

**Authors:** William D. Park, Barbara Okeke, Spencer Hobbs, Sneha Manikandan, Reem S. Ahmed, Lewis Frey, Uchenna Agbim, Mina M. Benjamin, SLU Transplant Rx Group

**Affiliations:** a Saint Louis University School of Medicine, St. Louis, Missouri, USA; b Department of Internal Medicine, SSM Health-Saint Louis University Hospital, St. Louis, Missouri, USA; c Division of Gastroenterology, Department of Internal Medicine, SSM Health-Saint Louis University Hospital, St. Louis, Missouri, USA; d Division of Cardiology, Department of Internal Medicine, SSM Health-Saint Louis University Hospital, St. Louis, Missouri, USA

**Keywords:** echocardiography, myocardial strain, predictive modeling, risk stratification

## Abstract

**Background:**

Cardiovascular events are a leading cause of mortality after liver transplantation (LT), and existing risk scores incompletely capture myocardial vulnerability.

**Objectives:**

The objective of the study was to evaluate whether pre-LT myocardial strain improves machine-learning risk stratification for major adverse cardiovascular events (MACE) following LT.

**Methods:**

We retrospectively studied 309 LT recipients with pretransplant echocardiography. Left ventricular global longitudinal strain, left atrial reservoir strain, and right ventricular free wall strain were measured. The primary endpoint was post-transplant MACE. Penalized Cox regression, random survival forest (RSF), and gradient boosting survival models were developed and benchmarked against coronary artery disease in LT and cardiovascular risk in orthotopic LT scores. Unsupervised clustering identified phenotypes.

**Results:**

The mean age was 55.8 ± 11.4 years, and 33.3% were females. Over a median 4.6 years, 65 patients experienced MACE (21.0%), with worse right ventricular free wall strain and left ventricular global longitudinal strain. RSF showed the highest discrimination (C-index 0.636; integrated Brier score 0.138). Adding multichamber strain produced small, statistically nonsignificant changes in discrimination (RSF ΔC-index +0.019; gradient boosting +0.028) and none in penalized Cox regression. The models did not outperform penalized Cox regression or the established LT-specific risk scores. By SHapley Additive exPlanations, strain ranked among the most important contributors. Clustering identified “cardiometabolic” and “hepatic” phenotypes.

**Conclusions:**

Machine-learning models showed only modest discrimination for post-transplant MACE and did not outperform conventional approaches or established LT risk scores; the incremental value of strain was not statistically significant. Nevertheless, multichamber strain consistently ranked among the strongest contributors in feature attribution, suggesting complementary biological information. These findings are hypothesis-generating and support further evaluation of strain within LT risk models in larger, externally validated cohorts.

Liver transplantation (LT) is the definitive treatment for end-stage liver disease. Cardiovascular complications remain the leading cause of nongraft-related mortality following LT with major adverse cardiovascular events (MACE) occurring in 10% to 25% of recipients within the first several years following LT.^1^, ^2^, ^3^ Patients with end-stage liver disease exhibit cardiovascular risk profiles that are comparable to or even exceed those of the general population with respect to traditional risk factors.^4^ In addition, the unique pathophysiology of end-stage liver disease contributes further to cardiovascular vulnerability through mechanisms such as systemic vasodilation, electrolyte and metabolic disturbances, concurrent renal impairment, malnutrition, and coagulation abnormalities.^5^

There is a significant heterogeneity between transplant programs when it comes to cardiovascular risk stratification. Cardiovascular risk stratification scores, such as coronary artery disease in LT (CAD-LT) and cardiovascular risk in orthotopic LT (CAR-OLT) scores, have been developed and validated but have shown modest performance in external validation studies. They rely largely on the identification of coronary artery disease, potentially missing other important markers of cardiac vulnerability, for example, pulmonary hypertension, subclinical myocardial systolic, and/or diastolic dysfunction.^6^, ^7^, ^8^ This gap in risk stratification contributes to suboptimal management and potentially preventable adverse outcomes.

Myocardial strain, typically measured using speckle-tracking echocardiography from 2-dimensional echocardiographic images, provides sensitive measures of myocardial systolic and diastolic function and has demonstrated prognostic value across a broad range of cardiovascular conditions.^9^ In transplant populations, strain abnormalities may reflect myocardial fibrosis, microvascular dysfunction, or subclinical systolic dysfunction that precede overt reductions in the left ventricular (LV) ejection fraction (LVEF).^10^^,^^11^ Prior studies have demonstrated that in LT recipients, LV global longitudinal strain (LV GLS) can identify patients at elevated risk even in the context of preserved LVEF.^12^ Left atrial reservoir strain (LASr), which is now incorporated into the 2025 guidelines update on the evaluation of diastolic function, reflects atrial remodeling and diastolic filling burden.^13^ LASr has been proposed in observational studies as a prognostic marker of several cardiovascular conditions, including in a LT population.^14^, ^15^, ^16^ Right ventricular (RV) free wall strain (RVSfw) may detect subclinical RV dysfunction with an incremental value beyond traditional RV function parameters. Similar findings have been observed in kidney transplant recipients.^17^ The value of RVSfw has not been explored in LT recipients who also have a high prevalence of right-sided afterload, myocardial fibrosis, and pulmonary hypertension.^18^

Machine learning approaches allow integration of multidomain clinical variables and may provide insight into hierarchical relationships among predictors of cardiovascular risk, including in transplant populations.^19^, ^20^, ^21^, ^22^ In addition, unsupervised clustering methods enable identification of phenotypic subgroups characterized by shared biological features across multiple domains.^23^ We hypothesized that multichamber strain would identify cardiovascular vulnerability beyond conventional variables in LT recipients. We further hypothesized that phenotype discovery across multidomain variables would identify biologically distinct subgroups associated with differential cardiovascular risk profiles.

## Methods

### Study design and patient population

We conducted a retrospective study of patients undergoing LT at SSM Health Saint Louis University Hospital between January 2014 and January 2024. The study was approved by the Saint Louis University Institutional Review Board with waiver of informed consent due to retrospective design (protocol #34091). Patients were eligible if they were aged ≥18 years and had an available pretransplant transthoracic echocardiogram study. Patients who did not follow up within our health system after LT were excluded. A total of 309 patients met these criteria. Clinical, laboratory, and echocardiographic variables were extracted from the electronic medical record and institutional imaging archives. Follow-up time was calculated as the interval in months from date of transplant to date of first MACE event, all-cause death, or last documented contact within our health system, whichever occurred first. Patients without MACE who remained alive were censored at their last recorded health-system encounter. The primary outcome was post-transplant MACE, defined as a composite of cardiovascular death, nonfatal myocardial infarction, stroke, heart failure hospitalization, ventricular arrhythmia, unstable angina hospitalization, and need for coronary revascularization. Outcomes were adjudicated by structured chart review using the prespecified criteria. Heart failure admissions were specifically adjudicated to confirm that heart failure was the primary reason for admission and treatment rather than an alternative diagnosis such as pneumonia or chronic obstructive pulmonary disease exacerbation. A table of all variable and outcome definitions is included in Supplemental Table 2.

### Echocardiography acquisition and strain analysis

Pretransplant transthoracic echocardiogram studies were obtained as part of routine pretransplant work-up. Our echocardiography lab uses GE HealthCare Vivid E95 ultrasound systems equipped with M5Sc-D phased array transducers (1.4-4.6 MHz). Standard 2-dimensional grayscale cine loops were obtained according to guideline-recommended acquisition protocols. Offline strain analysis was performed using vendor-independent software (TomTec Image Arena version 4.7; TomTec Imaging Systems GmbH). Twelve investigators underwent standardized training by the principal investigator (M.M.B.) before participation. Investigators were required to demonstrate ≥80% agreement with reference measurements on a predefined set of 10 studies before performing study measurements. Analysts were blinded to clinical characteristics and outcomes. Major manual contour modification was avoided to reduce interobserver variability. LV GLS was calculated from apical 2, 3, and 4-chamber views using automated speckle tracking. Segmental strain values were averaged to derive LV GLS. RVSfw was measured from a RV-focused apical 4-chamber view as the mean strain of basal, mid, and apical free wall segments. LASr was measured from apical 2- and 4-chamber views and averaged across views. Left atrial volumes were calculated using the biplane area-length method and indexed to body surface area. Absolute strain values are reported for interpretability, with lower values reflecting worse myocardial deformation.

### Feature selection and data preprocessing

Clinical, laboratory, and echocardiographic variables (386 variables) were collected. Variables with >30% missingness or near-zero variance (≥95% identical values) were excluded. Following data cleaning, 137 variables remained. Twenty-five variables were selected a priori based on clinical relevance and prior literature evaluating cardiovascular risk in a LT population. Selection of 25 variables was constrained by the events-per-variable ratio (EPV = 2.6, based on 65 events and 25 predictors), below the commonly recommended threshold of 10 to 20 for stable coefficient estimation. This motivated ridge-dominant penalization (L1 ratio fixed at 0.1) for the elastic net Cox model. Selected variables represented 4 domains:

• Demographic variables: age, sex, and body mass index (BMI).
• Hepatic variables: Model for End-Stage Liver Disease (MELD) score, albumin, estimated glomerular filtration rate (eGFR), metabolic dysfunction–associated steatotic liver disease, alcohol-associated liver disease, hepatitis C, and hepatocellular carcinoma.
• Medical comorbidities: hypertension, diabetes mellitus, coronary artery disease, atrial fibrillation, pulmonary hypertension, and smoking history (entered as 2 indicator variables—former and never smoking—with current smoking as the reference category).
• Echocardiographic variables: LVEF, LV GLS, LASr, RVSfw, RV fractional area change, tricuspid annular plane systolic velocity, RV index of myocardial performance, and tricuspid peak gradient.

Continuous variables were standardized using z-score normalization before modeling. Categorical variables were encoded using one-hot encoding. Before imputation, the missing completely at random assumption was formally evaluated using Little's test. To prevent imputation leakage into the cross-validation procedure, missing data were imputed using multivariate imputation by chained equations implemented via IterativeImputer (scikit-learn) within each outer cross-validation fold with the imputer fit exclusively on training-fold observations and applied to held-out test observations, ensuring test-fold information did not influence the imputation of training data. Imputation quality was assessed using convergence trace plots and density overlay plots comparing observed and imputed distributions per variable. Thirty imputed data sets were generated using 10 iterations each. Outcome variables (MACE status and follow-up time) were excluded from imputation. Physiologically plausible bounds were applied after pooling imputed datasets to limit implausible values, including MELD score (6-40), eGFR (2-120 mL/min/1.73 m^2^), and LV GLS (0% to 35%). Continuous variables were standardized after imputation.

### Supervised survival modeling

Three supervised survival modeling approaches were evaluated: elastic net–penalized Cox proportional hazards regression, random survival forest (RSF), and extreme gradient boosting (XGBoost) with survival objectives. Elastic net Cox regression was selected to provide a penalized regression comparator appropriate for moderate dimensionality and limited events per variable. RSF and gradient boosting methods were selected to capture nonlinear relationships and higher-order feature interactions. For established risk score benchmarking, the CAD-LT score^24^ and the CAR-OLT score^25^ were computed for each patient using the published point-based algorithms. Missing score components were set to zero (assuming absence). Scores were evaluated using the same framework applied to the other models. Model development used nested 5-fold cross-validation to reduce optimism bias. In the outer loop, the data set was partitioned into 5-folds, with each fold serving once as a held-out test set. In the inner loop, hyperparameters were optimized using cross-validation within the training folds. For elastic net Cox regression, alpha was tuned over 100 logarithmically-spaced values from 10^−6^ to 1.0 using inner 5-fold cross-validation with C-index as the selection criterion (Supplemental Figure 4); the L1 ratio was fixed at 0.1 a priori (ridge-dominant) given EPV = 2.6. For RSF, an exhaustive grid search was performed over 72 combinations: number of trees [300, 500, 750], minimum samples per leaf [10, 15, 20, 25], feature subsampling fraction [sqrt(p), 0.25, 0.33], bootstrap [True], and maximum subsampling fraction [0.80, 0.90]. For XGBoost with a survival:cox loss objective, 100 randomly sampled combinations were evaluated from a grid space of 4,374 combinations, covering: number of estimators [200, 300, 500], maximum tree depth [1, 2, 3], learning rate [0.02, 0.05, 0.10], subsample fraction [0.70, 0.80, 1.0], L2 regularization [0.5, 1.0, 5.0], minimum child weight [5, 10, 20], column subsampling fraction [0.70, 1.0], and gamma [0, 0.5, 1.0]. Maximum tree depth was capped at 3 and all hyperparameter ranges were determined empirically using the study data set before the final analysis (Supplemental Table 4). Model performance was summarized using pooled out-of-fold predictions. Model discrimination was evaluated using Harrell concordance index (C-index) as the primary metric. Secondary performance metrics included time-dependent area under the receiver operating characteristic curve (AUROC), area under precision-recall curve, and integrated Brier score. 95% CIs were estimated using 1,000 bootstrap resamples of pooled out-of-fold predictions. XGBoost integrated Brier score computed via Breslow’s baseline cumulative hazard estimator. Decision curve analysis was performed to evaluate net clinical benefit across a range of threshold probabilities. Risk scores were calibrated to probabilities using isotonic regression applied to out-of-fold predictions. The established risk scores (CAD-LT and CAR-OLT) were included on the same decision curve for direct comparison against treat-all and treat-none reference strategies. Calibration was assessed at a 3-year horizon by comparing each model’s predicted event probability (1 − Ŝ[36 months]; XGBoost survival derived from a Breslow baseline hazard) against the Kaplan-Meier observed cumulative incidence within quintiles of predicted risk. Overall observed-to-expected ratios were computed as the cohort Kaplan-Meier incidence divided by the mean predicted probability, with 95% CIs derived from the Kaplan-Meier interval; calibration slopes were estimated by regressing observed on predicted values across quintiles with 1,000-bootstrap 95% CIs. Given the modest class imbalance (21% event rate), no oversampling techniques were applied. Performance metrics robust to class imbalance, including area under precision-recall curve, were included.

### Competing risk analysis

To evaluate whether non-MACE mortality operated as a meaningful competing risk for MACE, a formal sensitivity analysis was performed. Non-MACE mortality was defined as all-cause mortality minus cardiovascular death (extracted from the outcome database); 26 patients experienced non-MACE death without prior MACE and were treated as competing events. The competing risk event indicator was coded as 0 (censored), 1 (MACE), or 2 (non-MACE death). Nonparametric cumulative incidence was estimated using the Aalen-Johansen estimator. Regression-based competing risk models were fitted using the subdistribution hazard framework (Fine-Gray) via inverse probability of censoring weighting applied to both elastic net Cox regression and RSF. XGBoost was not included in the competing risk analysis because the inverse probability of censoring weighting scheme cannot be correctly applied to the survival:cox partial likelihood.

### Unsupervised phenotype discovery

Unsupervised clustering was performed to identify multidomain phenotypes using 9 continuous variables representing cardiac (LVEF, LV GLS, LASr, and RVSfw), demographic (age and BMI), hepatic (MELD score and albumin), and renal (eGFR) domains. In addition, a secondary echo-based phenotype analysis was performed using only the 3 multichamber strain variables (LV GLS, LASr, and RVSfw). Continuous variables were standardized before clustering. Dimensionality reduction was performed using principal component analysis (PCA), retaining components explaining ≥80% of variance. Optimal cluster number was determined using silhouette score. To facilitate reproducibility, k-means cluster centroids are reported in both PCA space (the mathematical cluster centers defining the solution, enabling assignment of new patients via nearest-centroid classification) and original variable space (back-transformed means, SDs, and IQRs per clustering variable per cluster), along with PCA component loadings, in Supplemental Table 3. Cluster stability was evaluated using 200 bootstrap resamples with adjusted Rand index as the stability metric.

Cluster characteristics were compared using the Kruskal-Wallis test for continuous variables and the chi-square test for categorical variables. To assess incremental predictive value, cluster membership was added as an additional feature to the baseline data set and model performance metrics were recomputed using identical nested cross-validation procedures.

### Model interpretability

Feature attribution was assessed using SHapley Additive exPlanations (SHAP) values to quantify the relative contribution of individual variables to predicted risk. SHAP beeswarm plot was generated for the top performing model.

### Statistical analysis

All analyses were conducted in Python (version 3.12.10) using scikit-survival (0.27), scikit-learn (1.8), XGBoost (3.2), SHAP (0.51.0), and standard scientific computing libraries. Two-sided *P* values <0.05 were considered statistically significant. This study was conducted and reported in accordance with the TRIPOD + AI (Transparent Reporting of a multivariable prediction model for Individual Prognosis Or Diagnosis Artificial Intelligence)^26^ and PRIME (Proposed Requirements for Cardiovascular Imaging-Related Machine Learning Evaluation) guidelines^27^ with the checklist provided in Supplemental Material 1. Analysis code is available in Supplemental Material 2.

### Sex-based analysis

Sex was included as a biological variable in all predictive models and is reported in baseline characteristics (Table 1). Sex was not independently associated with MACE in univariate comparisons or feature attribution analyses. Gender identity was not systematically available in this retrospective dataset.

**Table 1:** Baseline Characteristics

	Total (N = 309)	MACE (n = 65)	No MACE (n = 244)	*P* Value
Age at transplant, y	55.8 ± 11.4	60.0 ± 8.7	54.7 ± 11.8	**0.001**
Follow-up time, y	4.6 [2.3-7.2]	1.7 [0.3-4.2]	5.2 [2.8-7.5]	**<0.001**
Male	206 (66.7%)	41 (63.1%)	165 (67.6%)	0.587
Race				0.474
White	278 (90.0%)	58 (89.2%)	220 (90.2%)	
Black	23 (7.4%)	4 (6.2%)	19 (7.8%)	
Other	8 (2.6%)	3 (4.6%)	5 (2.0%)	
BMI (kg/m^2^)	28.5 ± 5.0	28.7 ± 5.0	28.4 ± 4.9	0.712
Smoking history				0.061
Former	161 (52.1%)	42 (64.6%)	119 (48.8%)	
Current	14 (4.5%)	1 (1.5%)	13 (5.3%)	
Albumin (g/dL)	3.0 ± 0.7	2.9 ± 0.6	3.0 ± 0.7	0.511
MELD score	20.7 ± 8.9	18.9 ± 7.2	21.2 ± 9.2	0.109
eGFR (mL/min/1.73 m^2^)	54.5 ± 21.1	51.3 ± 17.5	55.5 ± 22.0	0.173
ESLD etiology				
MASLD	75 (24.3%)	25 (38.5%)	50 (20.5%)	**0.005**
Alcohol-associated liver disease	129 (41.7%)	31 (47.7%)	98 (40.2%)	0.341
Hepatitis C	65 (21.0%)	14 (21.5%)	51 (20.9%)	1.000
Hepatocellular carcinoma	59 (19.1%)	11 (16.9%)	48 (19.7%)	0.746
Cardiovascular comorbidities				
Hypertension	181 (58.6%)	45 (69.2%)	136 (55.7%)	0.069
Diabetes mellitus	96 (31.1%)	34 (52.3%)	62 (25.4%)	**<0.001**
Coronary artery disease	65 (21.0%)	25 (38.5%)	40 (16.4%)	**<0.001**
Atrial fibrillation	35 (11.3%)	13 (20.0%)	22 (9.0%)	**0.024**
Pulmonary hypertension	72 (23.3%)	22 (33.8%)	50 (20.5%)	**0.036**

Values are mean ± SD, median [IQR], or n (%). *P* values from 2-sample *t*-test (continuous) or chi-square test (categorical). Significant *P* values are **bold**.

BMI = body mass index; eGFR = estimated glomerular filtration rate; ESLD = end-stage liver disease; MACE = major adverse cardiovascular event; MASLD = metabolic dysfunction-associated steatotic liver disease; MELD = Model for End-Stage Liver Disease.

## Results

### Imputation quality

Little test rejected the missing-completely-at-random hypothesis (chi-square = 5,963.2, df = 877; *P* < 0.001), indicating that the data were not missing completely at random. A missing-at-random mechanism was therefore assumed, under which multiple imputation by chained equations provides valid estimates. Per-variable missingness rates are reported in Supplemental Table 1 and Supplemental Figure 3. Imputation quality assessed via convergence trace plots and density overlay plots (Supplemental Figure 1) demonstrated stable, flat trajectories of the mean imputed value across iterations for all imputed variables and close correspondence between observed and imputed distributions, supporting the plausibility of the imputed values. Among model features with missing data, missingness ranged from 0.6% to 29.4% (median 6.2%); for the 3 strain variables it was 21.0% (LASr), 21.4% (RVSfw), and 24.9% (LV GLS). Imputation was performed within each outer cross-validation fold (fit on training observations only), preventing leakage of held-out information.

### Study population

Among 309 LT recipients included in the analysis (mean age 55.8 ± 11.4 years; 33.3% females), 65 patients experienced at least 1 MACE event, comprising 96 total events across all components during a median follow-up of 4.6 (2.3-7.2) years, corresponding to an event rate of 21.0% (Table 1, Table 2, Table 3). The Kaplan-Meier estimated cumulative incidence of MACE was 8.9% (95% CI: 6.2%-12.7%) at 1 year, 14.0% (95% CI: 10.5%=-18.5%) at 3 years, and 18.7% (95% CI: 14.5%-24.0%) at 5 years. Heart failure hospitalization (28 of 96, 29.2%), stroke (18 of 96, 18.8%), and myocardial infarction (15 of 96, 15.6%) were the most frequent components of MACE (Table 3). Patients experiencing MACE were older (60.0 ± 8.7 years vs 54.7 ± 11.8 years; *P* = 0.001) and had statistically significant greater prevalence of diabetes mellitus (52.3% vs 25.4%; *P* < 0.001), coronary artery disease (38.5% vs 16.4%; *P* < 0.001), atrial fibrillation (20.0% vs 9.0%; *P* = 0.024), and pulmonary hypertension (33.8% vs 20.5%; *P* = 0.036). MELD score, albumin, BMI, and eGFR were not significantly different between groups (Table 1). LV systolic function was preserved overall (mean LVEF 65.8% ± 9.3%) and was not significantly different between groups. LV end-systolic and end-diastolic volume indices were modestly higher among patients with MACE (Table 2). Markers of myocardial deformation differed between groups, including significantly lower LV GLS (18.4% ± 3.8% vs 19.8% ± 4.4%; *P* = 0.045) and lower RVSfw (22.4% ± 6.9% vs 24.8% ± 7.4%; *P* = 0.034) among patients with MACE (Table 2). LASr demonstrated a trend toward lower values among patients with MACE (34.6% ± 10.0% vs 38.2% ± 12.1%; *P* = 0.053). Conventional RV functional indices including RV fractional area change, tricuspid annular plane systolic velocity, and RV index of myocardial performance were not significantly different between groups.

**Table 2:** Echocardiographic Parameters

	Total (N = 309)	MACE (n = 65)	No MACE (n = 244)	*P* Value
LV end systolic volume index (mL/m^2^)	21.8 ± 10.6	26.1 ± 10.6	20.9 ± 10.4	**0.013**
LV end diastolic volume index (mL/m^2^)	67.9 ± 23.0	74.5 ± 20.2	66.6 ± 23.4	**0.037**
LV ejection fraction (%)	65.8 ± 9.3	63.8 ± 8.1	66.4 ± 9.6	0.063
RVFAC (%)	41.5 ± 9.3	41.7 ± 9.0	41.5 ± 9.5	0.872
S' (cm/s)	16.4 ± 4.3	16.1 ± 5.4	16.4 ± 3.9	0.541
RIMP	0.31 [0.23-0.40]	0.32 [0.24-0.37]	0.31 [0.22-0.41]	0.847
Left atrial volume (mL)	64.9 ± 34.4	65.8 ± 36.6	64.4 ± 33.9	0.969
Left atrial volume index (mL/m^2^)	32.5 ± 16.4	32.7 ± 16.6	32.5 ± 16.4	0.983
Valvular disease severity (%)				
Mitral regurgitation severity				0.323
Mild	88 (30.3)	14 (23.3)	74 (32.2)	
Moderate or severe	1 (0.3)	1 (1.7)	0	
Tricuspid regurgitation severity				0.702
Mild	125 (43.4)	24 (40.7)	101 (44.1)	
Moderate or severe	4 (1.2)	1 (1.7)	3 (1.3)	
Aortic regurgitation severity				0.901
Mild	16 (5.6)	3 (4.9)	13 (5.8)	
Moderate or severe	2 (0.6)	1 (1.6)	1 (0.4)	
Aortic stenosis severity				0.477
Mild	8 (2.8)	2 (3.3)	6 (2.7)	
Moderate or severe	2 (0.6)	1 (1.7)	1 (0.4)	
LV global longitudinal strain (%)	19.5 ± 4.3	18.4 ± 3.8	19.8 ± 4.4	**0.045**
Right ventricular free wall strain (%)	24.3 ± 7.4	22.4 ± 6.9	24.8 ± 7.4	**0.034**
Left atrial reservoir strain (%)	37.4 ± 11.8	34.6 ± 10.0	38.2 ± 12.1	0.053

Values are mean ± SD, median [IQR], n (%). *P* values from 2-sample *t*-test (continuous) or Mann-Whitney *U* test (ordinal severity). Significant *P* values are **bold**.

LV = left ventricle; RIMP = right ventricular index of myocardial performance; RVFAC = right ventricular fractional area change; S' = tricuspid annular plane systolic velocity; other abbreviation as in Table 1.

**Table 3:** Major Adverse Cardiovascular Events (N = 96)

Cardiovascular death	8 (8.3)
Nonfatal myocardial infarction	15 (15.6)
Unstable angina hospitalization	6 (6.2)
Need for revascularization	9 (9.3)
Heart failure hospitalization	28 (29.2)
Major ventricular arrhythmia	12 (12.5)
Stroke	18 (18.8)

Values are n (%).

### Multivariable survival modeling

**Figure 1:**
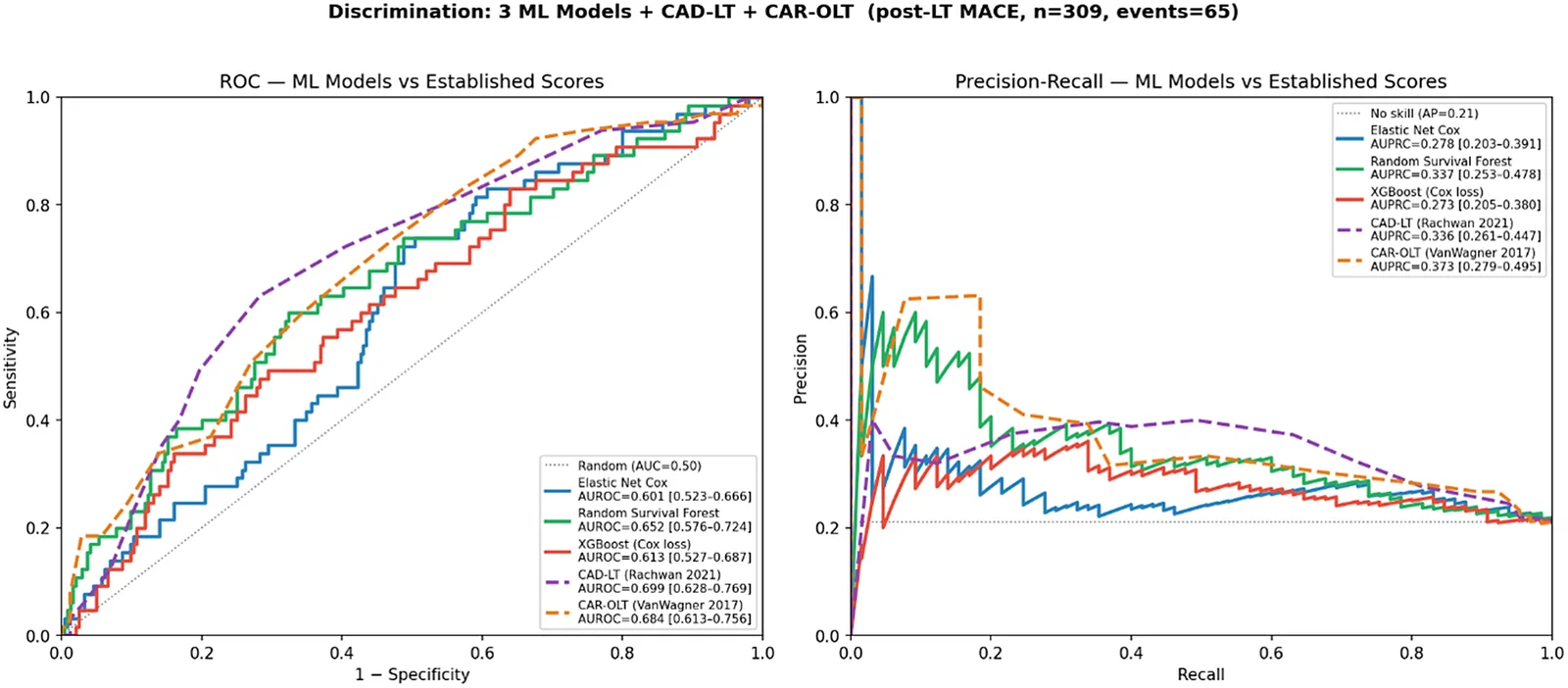
Discrimination of Machine Learning Models vs Historical Models for Prediction of Post-transplant Major Adverse Cardiovascular Events AP = average precision; AUC = area under the receiver operating characteristic curve; AUPRC = area under the precision-recall curve; CAD-LT = coronary artery disease in liver transplant score; CAR-OLT = cardiovascular risk in orthotopic liver transplantation score; LT = liver transplant; MACE = major adverse cardiovascular event; ML = machine learning; ROC = receiver operating characteristic curve; XGBoost = extreme gradient boosting.

**Figure 2:**
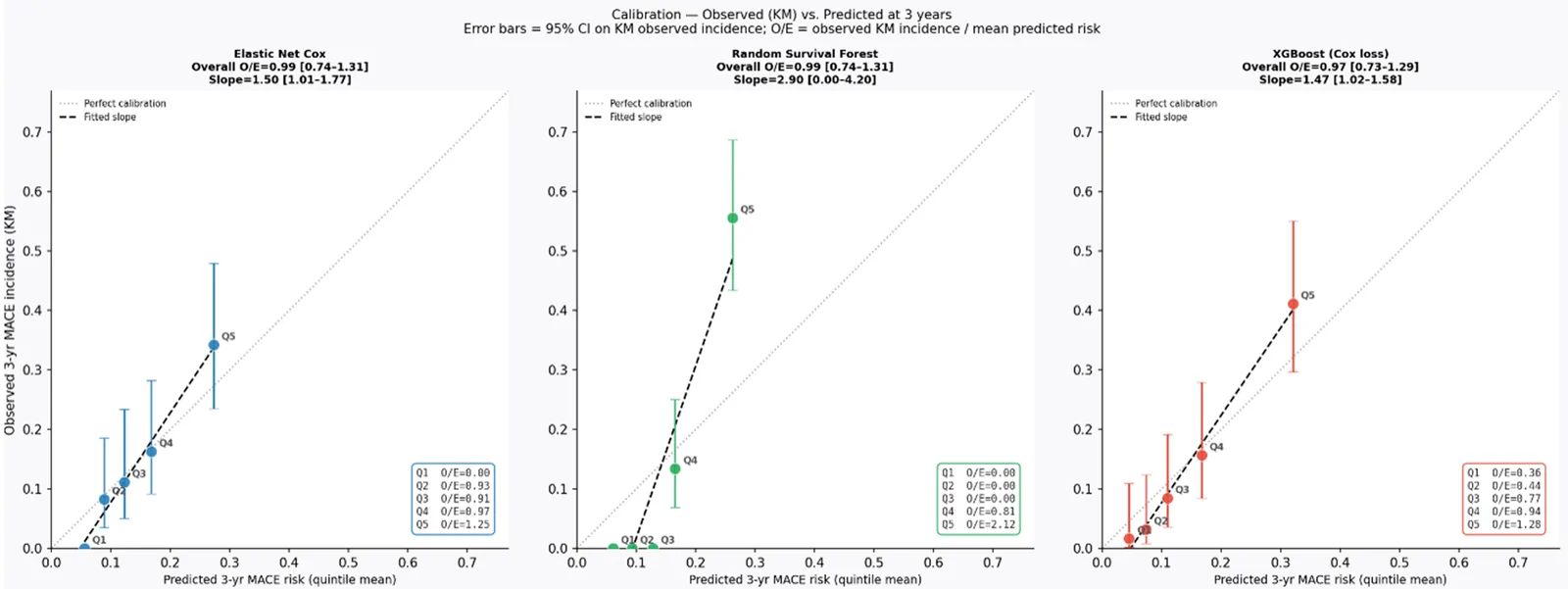
Calibration of Predicted vs Observed 3-Year Major Adverse Cardiovascular Event Risk KM = Kaplan-Meier; O/E = observed-to-expected; other abbreviations as in Figure 1.

**Figure 3:**
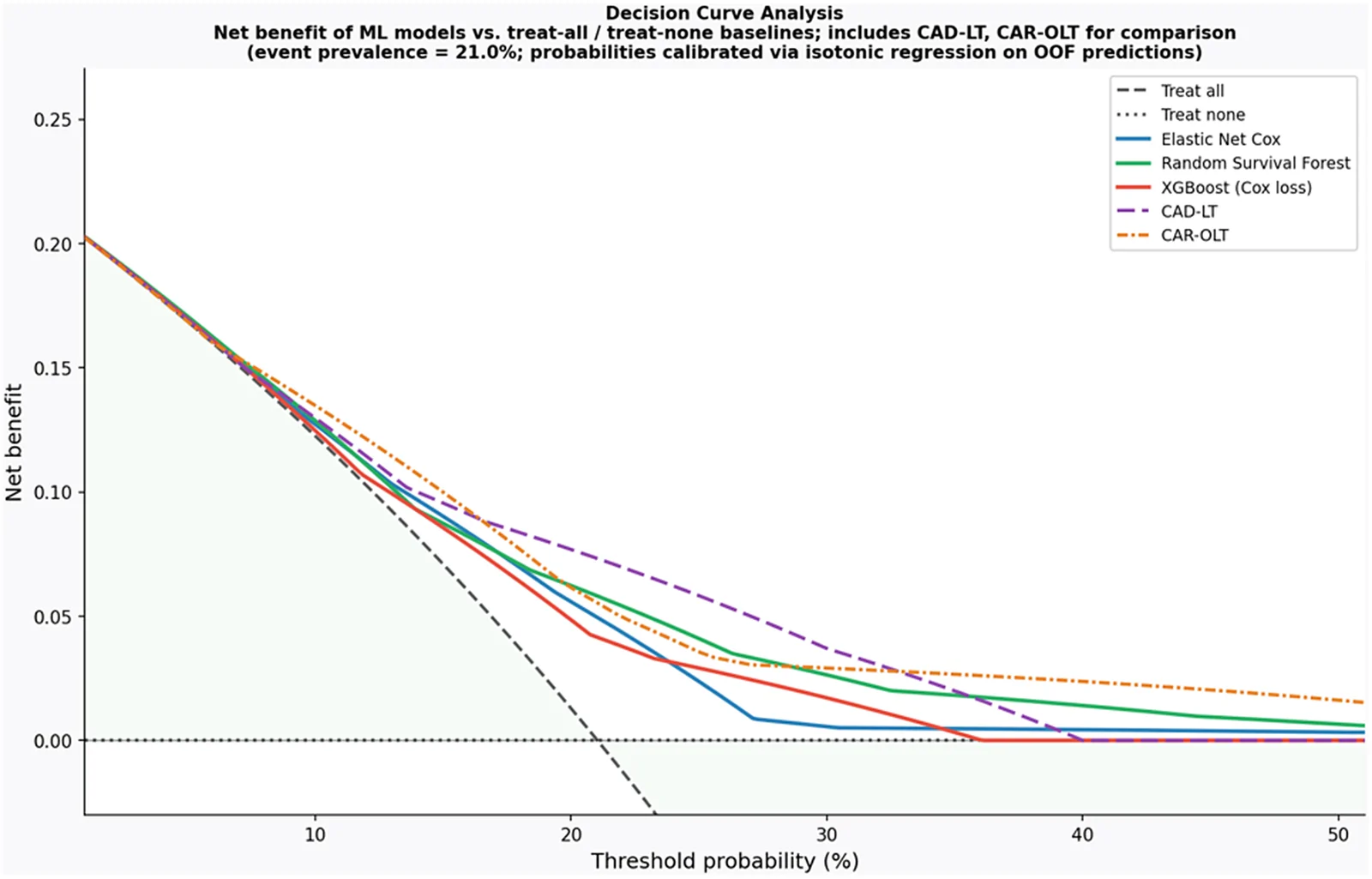
Decision Curve Analysis Showing Net Clinical Benefit Across Threshold Probabilities OOF = out-of-fold; other abbreviations as in Figure 1.

**Figure 4:**
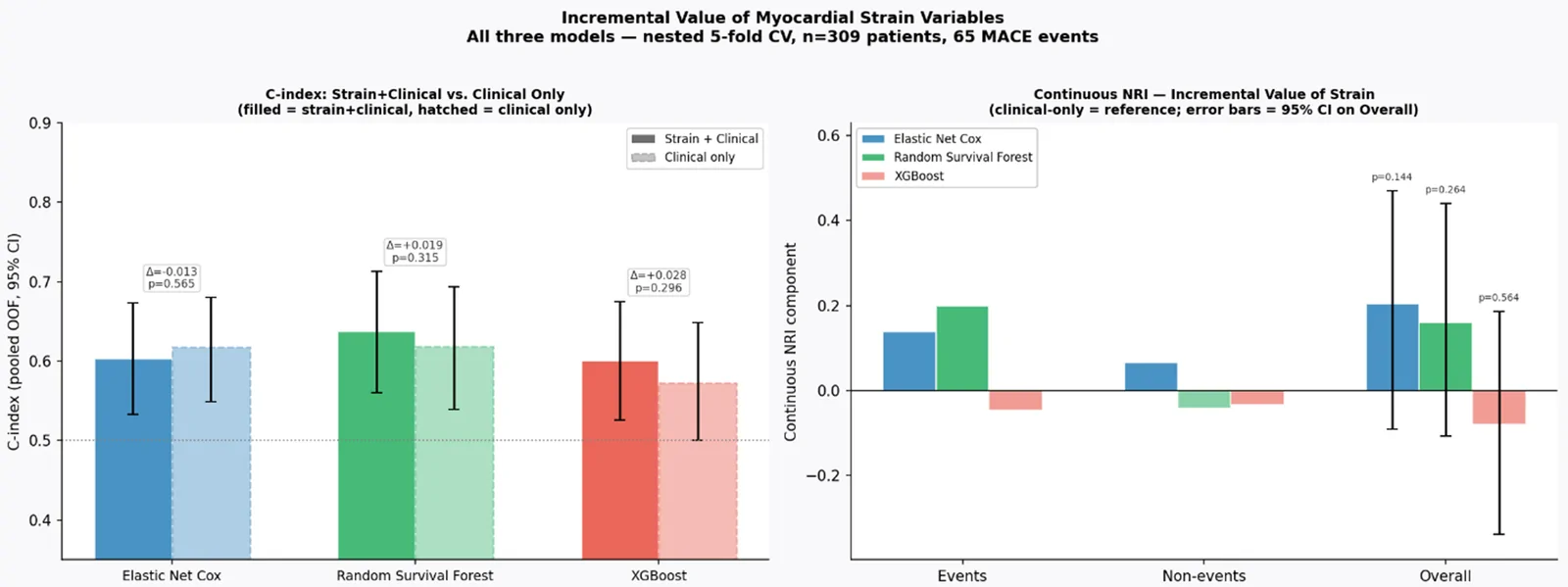
Incremental Discrimination of Multichamber Strain Over Clinical Variables Alone CV = cross-validation; NRI = net reclassification improvement; other abbreviations as in Figures 1 and 3.

Across supervised survival modeling approaches, discrimination for prediction of post-transplant MACE was modest but consistent across outer cross-validation folds (Table 4). RSF demonstrated the highest discrimination (C-index 0.636; 95% CI: 0.560-0.707), followed by elastic net Cox regression (C-index 0.603; 95% CI: 0.533-0.670) and XGBoost (C-index 0.599; 95% CI: 0.524-0.671) (Table 4). AUROC analysis showed similar performance patterns (Figure 1). Precision-recall analysis demonstrated average precision values of 0.360 (95% CI: 0.253-0.478) for RSF, 0.291 (95% CI: 0.203-0.387) for elastic net Cox regression, and 0.290 (95% CI: 0.205-0.380) for XGBoost. Observed-to-expected ratios were close to unity for all 3 models (elastic net Cox 0.99 [95% CI: 0.74-1.31]; RSF 0.99 [95% CI: 0.74-1.31]; XGBoost 0.98 [95% CI: 0.73-1.29]), with calibration slopes of 1.50, 2.90, and 1.47, respectively (Figure 2, Supplemental Table 5), indicating adequate calibration-in-the-large without systematic overprediction or underprediction. Decision curve analysis demonstrated net clinical benefit of the models across a range of threshold probabilities, comparable to the established risk scores (Figure 3). To evaluate the independent incremental contribution of myocardial strain, all 3 models were refitted using clinical variables only (excluding LV GLS, LASr, and RVSfw). The addition of multichamber strain modestly increased discrimination in the 2 ensemble models (RSF ΔC-index +0.019 [95% CI: −0.017 to +0.058]; XGBoost +0.028 [95% CI: −0.023 to +0.077]) and was essentially neutral in penalized Cox regression (−0.013 [95% CI: −0.055 to +0.029]); none reached statistical significance (permutation *P* = 0.315, 0.296, and 0.565, respectively) (Figure 4). Continuous net reclassification improvement was positive for Cox (+0.204 [95% CI: −0.091 to +0.469]; *P* = 0.144) and RSF (+0.159 [95% CI: −0.107 to +0.440]; events +0.200, nonevents −0.041; *P* = 0.264), and slightly negative for XGBoost (−0.079; *P* = 0.564). Thus, although strain did not significantly improve overall discrimination, its contribution was directionally positive for the models and it ranked among the leading variables in feature attribution (see SHAP analysis). Integrated Brier scores with 95% CIs were 0.138 [0.115–0.161] (RSF), 0.145 [0.121–0.170] (elastic net Cox), and 0.157 [0.129–0.187] (XGBoost) (Table 4).

**Table 4:** Machine Learning Models Performance

	C-Index [95% CI]	AUROC [95% CI]	AUPRC [95% CI]	Integrated Brier Score [95% CI]
Elastic Net Cox Regression	0.603 [0.533–0.670]	0.601 [0.523–0.666]	0.291 [0.203-0.387]	0.145 [0.121-0.170]
RSF	0.636 [0.560–0.707]	0.652 [0.576–0.724]	0.360 [0.253-0.478]	0.138 [0.115-0.161]
XGBoost	0.599 [0.524–0.671]	0.613 [0.527–0.687]	0.290 [0.205-0.380]	0.157 [0.129-0.187]
CAD-LT	0.663 [0.587-0.736]	0.699 [0.628–0.769]	0.336 [0.261–0.447]	N/A
CAR-OLT	0.670 [0.601–0.742]	0.684 [0.613–0.756]	0.374 [0.279–0.495]	N/A

XGBoost integrated Brier score computed via Breslow’s baseline cumulative hazard estimator.

AUPRC = area under the precision-recall curve; AUROC = area under the receiver operating characteristic curve; CAD-LT = coronary artery disease in liver transplant score; CAR-OLT = cardiovascular risk in orthotopic liver transplantation score; N/A = not applicable; RSF = random survival forest; XGBoost = extreme gradient boosting.

### Established score comparison to CAD-LT and CAR-OLT

For contextual benchmarking, CAD-LT demonstrated a C-index of 0.663 (95% CI: 0.587-0.736) and AUROC of 0.699 (95% CI: 0.628-0.769). CAR-OLT demonstrated a C-index of 0.670 (95% CI: 0.601-0.742) and AUROC of 0.684 (95% CI: 0.613-0.756) (Table 4, Figure 1). Discrimination of both established scores was comparable to—indeed numerically slightly higher than—the machine-learning models, reinforcing that the contribution of the present analysis is mechanistic rather than a gain in predictive accuracy. The respiratory-failure component of CAR-OLT was missing in 118 of 309 (38.2%) and scored as absent.

### Competing risk analysis

In a prespecified sensitivity analysis addressing competing risks from noncardiovascular mortality (n = 26 competing events, 8.4% of the cohort), the Aalen-Johansen cumulative incidence of MACE was 8.8%, 13.3%, and 18.2% at 12, 36, and 60 months, closely tracking the 1 − Kaplan-Meier estimates (8.9%, 14.0%, and 18.7%), indicating only modest competing risk bias at the cohort level. Fine-Gray subdistribution Cox regression improved discrimination relative to standard elastic net Cox (C-index 0.655 [95% CI: 0.585-0.713] vs 0.617 [95% CI: 0.566-0.673]; ΔC = +0.039), whereas competing-risk RSF was essentially unchanged from standard RSF (0.660 [95% CI: 0.589-0.737] vs 0.656 [95% CI: 0.587-0.736]; ΔC = +0.003). Note that these C-indices were estimated on the full cohort using inverse probability of censoring weighting rather than nested cross-validation, and are therefore not directly comparable to the primary out-of-fold estimates in Table 4. Accounting for the competing risk had a meaningful effect for the linear model but minimal effect for the best-performing ensemble model (Supplemental Figure 2). The standard-model C-indices reported in this competing-risk sensitivity analysis are estimated within the complete-case Fine-Gray/IPCW framework and are therefore not directly comparable to the pooled out-of-fold estimates in Table 4.

### Multidomain phenotype identification

**Figure 5:**
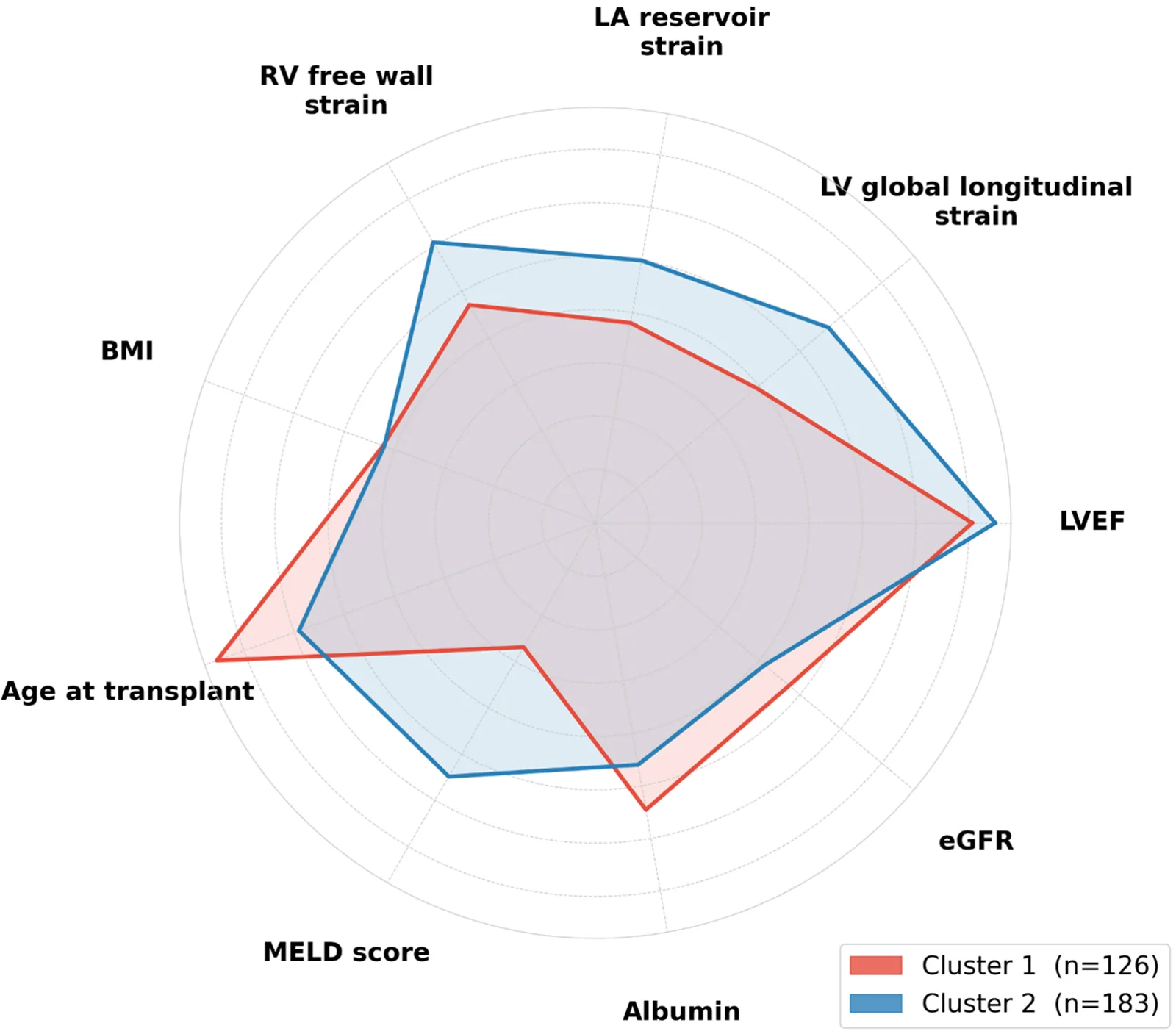
Multidomain Phenotype Characterization by Unsupervised Clustering BMI = body mass index; eGFR = estimated glomerular filtration rate; LA = left atrial; LV = left ventricular; LVEF = left ventricular ejection fraction; MELD = Model for End-Stage Liver Disease; RV = right ventricular.

Unsupervised clustering using multidomain continuous variables identified 2 phenotypes as the optimal solution based on silhouette score (Figure 5) and bootstrap cluster stability (Supplemental Figure 5). PCA retained 6 components explaining 82.9% of total variance, and bootstrap validation demonstrated stable clustering with an adjusted Rand index of 0.712 (95% CI: 0.267-0.936). Cluster 1 (n = 126 of 309, 40.8%; the “cardiometabolic” phenotype) demonstrated a 1.6-fold higher incidence of MACE than cluster 2 (n = 183 of 309, 59.2%; the “hepatic” phenotype) (27.0% vs 16.9%; *P* = 0.047). Cluster 1 was characterized by older age, lower myocardial strain, higher prevalence of diabetes mellitus and coronary artery disease, and lower MELD scores and higher albumin compared with cluster 2 (Figure 5, Table 5). Cluster 1 also had a higher prevalence of hepatocellular carcinoma and hepatitis C, consistent with an older, well-compensated population transplanted at lower MELD.

**Table 5:** Cluster Phenotype Characterization

	Cluster 1 (n = 126)	Cluster 2 (n = 183)	*P* Value
Age at transplant, y	61.2 ± 8.0	52.1 ± 11.9	**<0.001**
Male	91 (72.2%)	115 (62.8%)	0.110
Body mass index (kg/m^2^)	28.5 ± 5.0	28.4 ± 4.9	0.760
Albumin (g/dL)	3.2 ± 0.7	2.8 ± 0.7	**<0.001**
MELD score	15.1 ± 5.4	24.7 ± 6.5	**<0.001**
eGFR (mL/min/1.73 m^2^)	57.9 ± 15.8	50.9 ± 21.2	**<0.001**
End stage liver disease etiology (%)			
MASLD	37 (29.4)	38 (20.8)	0.110
Alcohol-associated liver disease	37 (29.4)	92 (50.3)	**<0.001**
Hepatitis C	40 (31.7)	25 (13.7)	**<0.001**
Hepatocellular carcinoma	43 (34.1)	16 (8.7)	**<0.001**
Cardiovascular comorbidities (%)			
Hypertension	82 (65.1)	105 (57.4)	0.214
Diabetes mellitus	50 (39.7)	46 (25.1)	**0.010**
Coronary artery disease	44 (34.9)	21 (11.5)	**<0.001**
Atrial fibrillation	20 (15.9)	15 (8.2)	0.056
Pulmonary hypertension	29 (23.0)	43 (23.5)	1.000
Smoking history	67 (53.2)	94 (51.4)	0.844
Echocardiographic parameters (%)			
Left ventricular ejection fraction	63.7 ± 10.0	67.4 ± 8.0	**<0.001**
Left atrial reservoir strain	32.0 ± 8.9	41.2 ± 9.9	**<0.001**
Right ventricular free wall strain	21.0 ± 6.2	26.6 ± 5.9	**<0.001**
Left ventricular global longitudinal strain	17.0 ± 3.1	21.2 ± 3.3	**<0.001**
Major adverse cardiovascular events	34 (27.0%)	31 (16.9%)	**0.047**

Values are mean ± SD or n (%). *P* values from 2-sample t-test (continuous) or chi-square/Fisher exact test (categorical). Statistically significant *P* values are **bold**.

Abbreviations as in Table 1.

### Echo subphenotype identification

A secondary echo-based subphenotype analysis using only the 3 multichamber strain variables (LV GLS, LASr, and RVSfw) identified 4 distinct strain subphenotypes (silhouette score = 0.267; adjusted Rand index = 0.589 [95% CI: 0.263-0.947]) (Supplemental Figure 6). MACE incidence differed significantly across subphenotypes (*P* = 0.009): the subphenotype with the worst deformation across all 3 chambers (lowest LV GLS, LASr, and RVSfw; oldest, with the highest prevalence of coronary artery disease) had the highest MACE rate (33.3%), whereas the most-preserved subphenotype had the lowest (9.4%). This strain-defined gradient paralleled the cardiometabolic phenotype identified in the multidomain analysis, suggesting that multichamber strain captures a substantial component of the same systemic cardiometabolic vulnerability. Given the modest sample size and event count, these subphenotype findings should be regarded as exploratory and hypothesis-generating.

### Relative importance of clinical and imaging features

**Figure 6:**
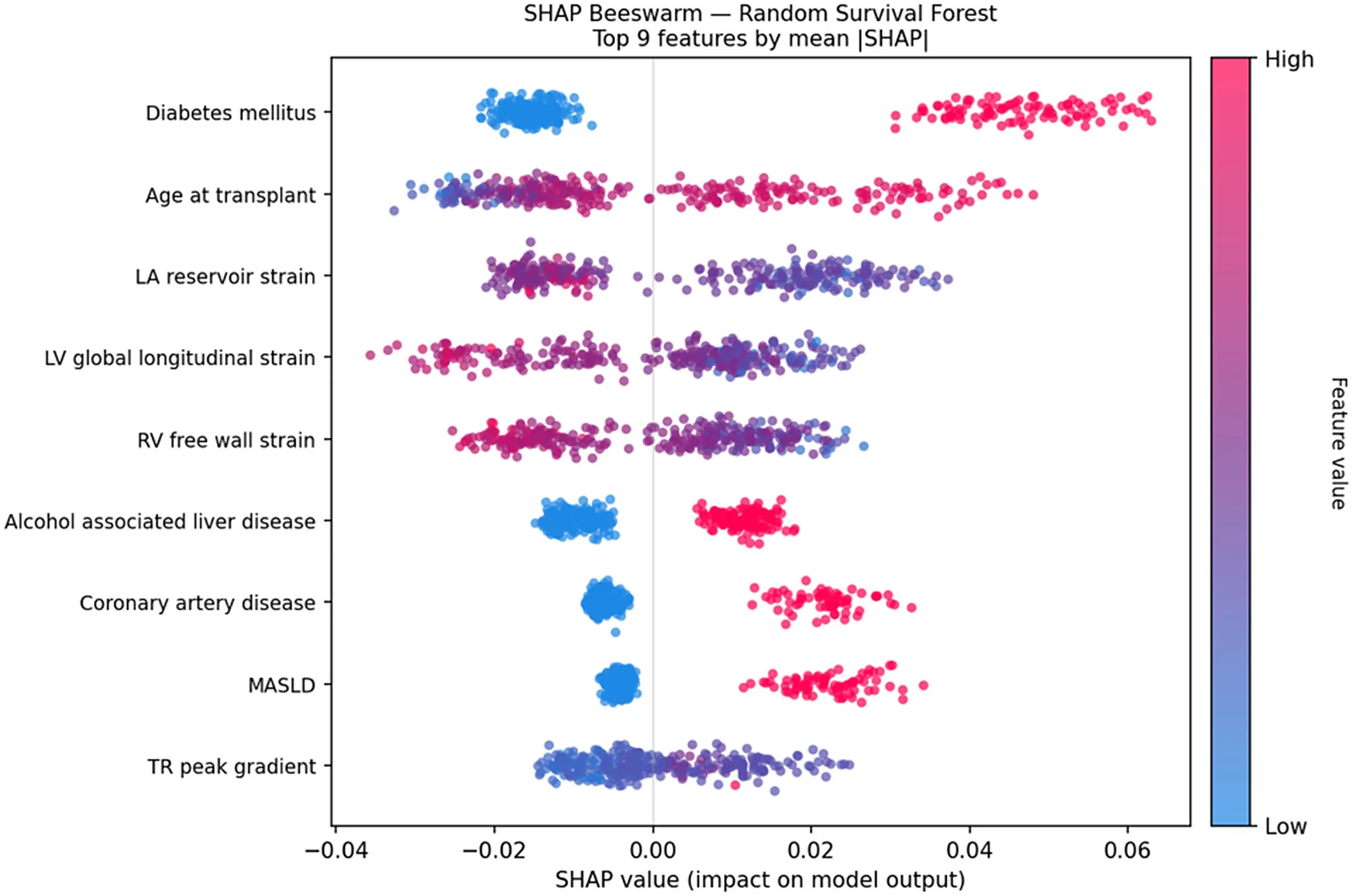
SHapley Additive exPlanations Beeswarm Plot Illustrating Relative Variable Importance MASLD = metabolic dysfunction-associated steatotic liver disease; SHAP = SHapley Additive exPlanations; TR = tricuspid regurgitation; other abbreviations as in Figure 5.

SHAP-based feature attribution demonstrated consistent ranking of predictors across models (Figure 6). Age and diabetes mellitus were among the strongest clinical contributors to predicted risk. Among echocardiographic variables, LV GLS, RVSfw, and LASr demonstrated strong contributions to MACE prediction with dependence plots demonstrating fairly monotonic relationships with cardiovascular risk. The phenotype classification did not significantly improve discrimination for MACE prediction. Adding cluster-phenotype membership as a predictor did not significantly improve discrimination for any model (elastic net Cox ΔC-index +0.008, *P* = 0.227; RSF +0.003, *P* = 0.720; XGBoost +0.011, *P* = 0.422; all DeLong p for AUROC ≥0.74) (Supplemental Figure 7, Supplemental Table 6). This supports the interpretation that the multidomain phenotype, although biologically informative, does not add independent predictive information beyond the constituent variables already in the models.

## Discussion

In this retrospective study of LT recipients, machine-learning models demonstrated only modest discrimination for post-transplant MACE and performed comparably to penalized Cox regression and to the established LT-specific risk scores; the incremental discrimination contributed by multichamber strain was small and did not reach statistical significance. The principal contribution of this study is therefore not improved prediction but the biological and phenotypic information conveyed by multichamber strain, which consistently ranked among the leading variables in feature attribution and may capture myocardial vulnerability not reflected in conventional clinical variables. Unsupervised multidomain clustering further identified a “cardiometabolic-predominant” phenotype characterized by relatively impaired strain across chambers and a higher burden of traditional cardiovascular risk factors. This phenotype was associated with significantly higher post-transplant MACE. However, the phenotype classification itself was not independently associated with MACE risk (Central Illustration).

**Central Illustration:**
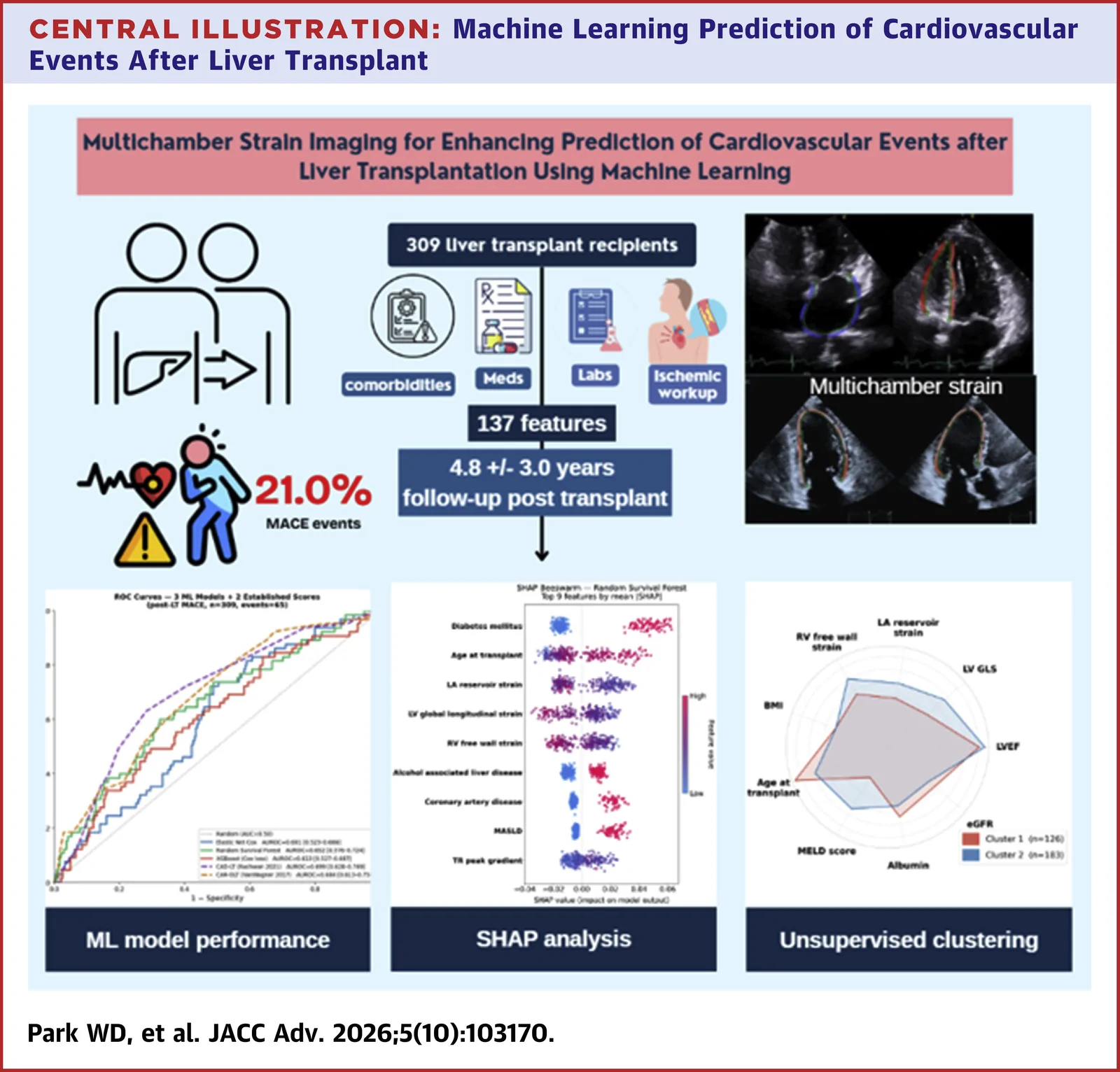
Machine Learning Prediction of Cardiovascular Events After Liver Transplant AUC = area under the receiver operating characteristic curve; AUPRC = area under the precision-recall curve; BMI = body mass index; CAD-LT = coronary artery disease in liver transplant score; CAR-OLT = cardiovascular risk in orthotopic liver transplantation score; MACE = major adverse cardiovascular event; MASLD = metabolic dysfunction-associated steatotic liver disease; MELD = Model for End-Stage Liver Disease; ML = machine learning; ROC = receiver operating characteristic curve; XGBoost = extreme gradient boosting; eGFR = estimated glomerular filtration rate; LA = left atrial; LV = left ventricular; LVEF = left ventricular ejection fraction; RV = right ventricular; SHAP = SHapley Additive exPlanations; TR = tricuspid regurgitation.

Our findings support the hypothesis that myocardial deformation imaging may detect subclinical myocardial dysfunction in LT recipients. Subtle changes in LV GLS may detect subclinical myocardial dysfunction even when the LVEF is preserved—or even hyperdynamic—from systemic vasodilation in end-stage liver disease patients. RVSfw may reflect pulmonary vascular remodeling, volume overload, and RV myocardial fibrosis—processes frequently observed in advanced liver disease and metabolic dysfunction. LASr likely reflects cumulative exposure to chronically elevated filling pressures and ventricular diastolic dysfunction, both of which are common in cirrhosis and cardiometabolic disease. The borderline association of LASr with MACE (*P* = 0.053) could reflect limited statistical power given the event count rather than absence of a biological effect; LASr nevertheless still ranked among the leading echocardiographic contributors in SHAP analysis, following LV GLS and RVSfw.

Interestingly, even though the differences in strain values between the patients who experienced MACE and those who did not were statistically significant, the mean strain values for both groups were within “normal” values per societal guideline statements.^28^^,^^29^ This observation has 2 implications. First, it suggests that in LT recipients myocardial strain may behave as a continuous marker of cardiovascular vulnerability rather than a dichotomous diagnostic threshold. Second, it implies that population-derived strain thresholds may underestimate risk in this population, and that continuously modeled or patient-referenced strain values may be more informative than fixed cutoffs. Consistent with this, no discrete strain threshold separating patients with and without MACE emerged from our data. Prospective work is needed to determine whether transplant-specific strain reference ranges improve discrimination.

The identification of a “cardiometabolic-predominant” phenotype, which demonstrated a substantially higher rate of MACE despite lower MELD scores, suggests that severity of liver disease alone does not fully capture the magnitude of cardiovascular vulnerability. These findings are consistent with emerging paradigms in cardiometabolic disease, in which systemic metabolic dysfunction contributes to myocardial injury through inflammatory, fibrotic, and microvascular mechanisms.^30^^,^^31^

Recent studies have explored machine learning approaches to prediction of cardiovascular outcomes following LT.^19^^,^^20^ Prior models have incorporated demographic, laboratory, and clinical variables using random forest, gradient boosting, or neural network approaches, demonstrating modest discrimination for post-transplant cardiovascular events. Our findings extend prior work by demonstrating that incorporation of multichamber strain measures may provide incremental biological insight, although overall model discrimination remained comparable to prior studies. This may be attributable in part to the modest sample size of the present cohort. In contrast to prior deep learning approaches that emphasize algorithmic performance, the present study aimed to use interpretable machine learning methods to characterize the relative importance of individual clinical and imaging variables that are often unavailable in large registry-based datasets. Importantly, the performance of machine learning models in the present study was comparable to penalized Cox regression, suggesting that the primary value of machine learning in this context may lie in variable prioritization and interaction detection rather than substantial gains in discrimination. These findings are consistent with prior cardiovascular machine learning studies demonstrating that gains in predictive performance may be modest when datasets are relatively small or when predictors are clinically interpretable.^32^, ^33^, ^34^ Notably, the ensemble methods did not significantly outperform penalized Cox regression, and both were comparable to—or modestly below—established point-based risk scores; differences in discrimination fell within overlapping CIs. The principal contribution of the machine-learning framework in this setting was therefore interpretability—consistent variable prioritization through SHAP and the ability to accommodate nonlinear effects (reflected in the positive incremental value of strain for the ensemble models but not for the linear model)—rather than improved predictive accuracy, a pattern expected when sample size and event counts are limited.

Our findings suggest that incorporation of myocardial deformation imaging may provide complementary information beyond traditional clinical risk scores. Furthermore, the identification of a “cardiometabolic-predominant” phenotype characterized by impaired multichamber strain and higher prevalence of diabetes and coronary artery disease suggests that myocardial deformation measures may serve as integrative markers of systemic cardiometabolic burden. The cardiometabolic-predominant phenotype more likely reflects systemic cardiometabolic disease rather than cirrhotic cardiomyopathy: it was defined by older age, diabetes, and coronary artery disease together with impaired multichamber strain, yet occurred at lower MELD scores, arguing against advanced hepatic dysfunction as the principal driver. Clinically, this implies that a subset of LT candidates carries cardiovascular risk attributable to metabolic derangement rather than primary hepatic disease; those may benefit from intensified cardiometabolic risk-factor modification during transplant evaluation and following LT. The phenotypes were reproducible on bootstrap resampling (adjusted Rand index 0.712), and a strain-only subphenotype analysis recovered a concordant high-risk, pan-chamber-impaired group (MACE 33.3% vs 9.4% in the most-preserved subgroup, *P* = 0.009), supporting robustness of the clustering. Because clustering depends on the choice of variables and distance metric, alternative algorithms could yield different structures; these phenotypes should be regarded as hypothesis-generating. Future studies should further explore whether imaging biomarkers may enhance pretransplant risk stratification frameworks that currently rely predominantly on clinical variables, especially coronary artery disease.

### Study Limitations

Several limitations should be considered when interpreting our study findings. First, this was a single-center retrospective study with a modest sample size, which may limit generalizability. Ascertainment of outcomes relied on chart review, and even though we included only patients who followed up at our institution, we could not completely rule out the possibility that MACE events were undercounted if patients were hospitalized at outside institutions not captured in our medical records. Strain measurements, although standardized, are inherently operator and software dependent. Missingness among the strain variables was also substantial (21% to 25%), reflecting the practical challenge that adequate-quality strain is not obtainable in all pretransplant studies; image quality is frequently limited in end-stage liver disease by body habitus, ascites, and tachycardia, which may constrain the real-world availability of strain in transplant populations. Although imputation was performed rigorously within cross-validation folds, this degree of missingness may nevertheless attenuate observed associations. In addition, we did not assess serial changes in strain before or after LT. Variables with near-zero variance or high missingness were removed during preprocessing; although this also reduces overfitting and instability under a low events-per-variable ratio, it may have excluded low-prevalence variables with genuine prognostic relevance. Clustering methods rely on assumptions regarding distance metrics and variable selection, and alternative clustering approaches may yield different phenotype structures. In addition, causality cannot be inferred from observational data, and myocardial deformation abnormalities may represent markers rather than mediators of cardiovascular risk. External validation will be essential before clinical use. Suitable validation cohorts could include multicenter transplant registries that link pretransplant echocardiographic strain to longitudinal outcomes; transportability across centers may be limited by differences in strain vendor software, case mix, and event ascertainment, underscoring the need for harmonized acquisition and outcome definitions. Finally, incremental discrimination from strain was modest and did not reach statistical significance, indicating that strain should be regarded as a contributory marker rather than a stand-alone discriminator in this cohort.

In conclusion, machine-learning methods did not improve discrimination beyond penalized Cox regression or established LT-specific risk scores, and the incremental value of strain was not statistically significant. The principal contribution of this work is thus mechanistic and phenotypic rather than a demonstrated gain in predictive performance. These findings should be regarded as hypothesis-generating and support further evaluation of multichamber strain within LT risk-stratification models in larger, externally validated cohorts.

Perspectives **COMPETENCY IN MEDICAL KNOWLEDGE:** In LT recipients, multichamber echocardiographic strain, such as LV GLS, RVSfw, and LASr may identify cardiovascular vulnerability beyond traditional metrics.

**COMPETENCY IN PATIENT CARE AND PROCEDURAL SKILLS:** Regular acquisition of multichamber strain during pretransplant echocardiography may potentially enhance cardiovascular phenotyping and—eventually—enhance individualized surveillance and preventative strategies.

**TRANSLATIONAL OUTLOOK:** Multichamber myocardial strain parameters may provide complementary information regarding myocardial vulnerability that is not fully captured by conventional measures such as LVEF. Incorporation of strain into pretransplant echocardiographic assessment may improve characterization of subclinical myocardial dysfunction in liver transplantcandidates. Further prospective studies are needed to determine whether integration of strain parameters into existing risk stratification frameworks improves prediction of posttransplant cardiovascular outcomes. Multidomain phenotyping incorporating cardiac, metabolic, hepatic, and renal variables may help identify clinically meaningful subgroups of transplant candidates with differing cardiovascular risk profiles. Although clustering did not significantly improve model discrimination in this study, phenotype-based approaches may facilitate improved biological understanding of cardiometabolic vulnerability in liver transplant populations. Future multicenter investigations are needed to evaluate whether phenotype-guided risk stratification may inform individualized surveillance strategies or targeted preventive interventions.

## Funding support and author disclosures

This study was funded by a grant from the Missouri Chapter of the American College of Cardiology (Grant-002620). The authors have reported that they have no relationships relevant to the contents of this paper to disclose.
