# Supplementary material for: Multichamber Myocardial Strain for Cardiovascular Risk Stratification in Liver Transplant Recipients Using Interpretable Machine Learning
Source: JACC Adv. 2026 Aug 26;5(10):103170. doi: 10.1016/j.jacadv.2026.103170 (PMC13544291; doi:10.1016/j.jacadv.2026.103170)
Supplement: Supplementary file 1 [file mmc1.docx]

Supplementary Table 1. Per Feature Missingness

| **Feature** | **Total (N=309)** | **MACE (n=65)** | **No MACE (n=244)** |
| --- | --- | --- | --- |
| Age at transplant | 0 (0.0%) | 0 (0.0%) | 0 (0.0%) |
| Left atrial reservoir strain | 65 (21.0%) | 13 (20.0%) | 52 (21.3%) |
| Right ventricular free wall strain | 66 (21.4%) | 12 (18.5%) | 54 (22.1%) |
| Left ventricular ejection fraction | 19 (6.1%) | 6 (9.2%) | 13 (5.3%) |
| Tricuspid regurgitation peak gradient | 67 (21.7%) | 16 (24.6%) | 51 (20.9%) |
| RVFAC | 51 (16.5%) | 10 (15.4%) | 41 (16.8%) |
| Left ventricular global longitudinal strain | 77 (24.9%) | 15 (23.1%) | 62 (25.4%) |
| S*'* | 20 (6.5%) | 4 (6.2%) | 16 (6.6%) |
| RIMP | 15 (4.9%) | 5 (7.7%) | 10 (4.1%) |
| Male sex | 0 (0.0%) | 0 (0.0%) | 0 (0.0%) |
| MASLD | 0 (0.0%) | 0 (0.0%) | 0 (0.0%) |
| Alcohol-associated liver disease | 0 (0.0%) | 0 (0.0%) | 0 (0.0%) |
| Hepatitis C | 0 (0.0%) | 0 (0.0%) | 0 (0.0%) |
| Hepatocellular Carcinoma | 0 (0.0%) | 0 (0.0%) | 0 (0.0%) |
| BMI | 4 (1.3%) | 1 (1.5%) | 3 (1.2%) |
| Albumin | 4 (1.3%) | 1 (1.5%) | 3 (1.2%) |
| MELD score | 91 (29.4%) | 19 (29.2%) | 72 (29.5%) |
| eGFR | 70 (22.7%) | 13 (20.0%) | 57 (23.4%) |
| Hypertension | 6 (1.9%) | 2 (3.1%) | 4 (1.6%) |
| Diabetes mellitus | 3 (1.0%) | 0 (0.0%) | 3 (1.2%) |
| Coronary artery disease | 6 (1.9%) | 0 (0.0%) | 6 (2.5%) |
| Former smoker | 0 (0.0%) | 0 (0.0%) | 0 (0.0%) |
| Non-smoker | 0 (0.0%) | 0 (0.0%) | 0 (0.0%) |
| Pulmonary hypertension | 2 (0.6%) | 0 (0.0%) | 2 (0.8%) |
| Atrial fibrillation | 6 (1.9%) | 1 (1.5%) | 5 (2.0%) |

*Variables listed as n (%). BMI = body mass index; eGFR = estimated glomerular filtration rate; MACE = major adverse cardiovascular event; MASLD = metabolic dysfunction-associated steatotic liver disease; MELD = Model for End-Stage Liver Disease; RIMP = right ventricular index of myocardial performance; RVFAC = right ventricular fractional area change; S' = tricuspid annular plane systolic velocity.*

Supplementary Table 2. Feature Data Dictionary

| **Feature** | **Type** | **Description** |
| --- | --- | --- |
| Age at transplant | Continuous | Patient age at the time of liver transplantation (years) |
| Left atrial reservoir strain | Continuous | Echocardiographic measure of left atrial reservoir function (%) |
| Right ventricular free wall strain | Continuous | Echocardiographic measure of right ventricular free wall longitudinal deformation (%) |
| Left ventricular ejection fraction | Continuous | Percentage of blood ejected from the left ventricle with each contraction (%) |
| Tricuspid regurgitation peak gradient | Continuous | Peak pressure gradient across the tricuspid valve measured by Doppler echocardiography (mmHg) |
| RVFAC | Continuous | Echocardiographic measure of right ventricular systolic function calculated as (end-diastolic area – end-systolic area) / end-diastolic area (%) |
| Left ventricular global longitudinal strain | Continuous | Speckle-tracking echocardiographic measure of left ventricular longitudinal deformation (%) |
| S' | Continuous | Tricuspid annular plane systolic velocity measured by tissue Doppler imaging (cm/s) |
| RIMP | Continuous | Right ventricular index of myocardial performance (Tei index); measure of combined right ventricular systolic and diastolic function |
| Male sex | Binary | Patient sex (1 = male, 0 = female) |
| MASLD | Binary | Metabolic dysfunction-associated steatotic liver disease as etiology of liver disease (1 = yes, 0 = no) |
| Alcohol-associated liver disease | Binary | Alcohol-associated liver disease as etiology of liver disease (1 = yes, 0 = no) |
| Hepatitis C | Binary | Hepatitis C virus infection as etiology of liver disease (1 = yes, 0 = no) |
| Hepatocellular carcinoma | Binary | Hepatocellular carcinoma as indication for transplantation (1 = yes, 0 = no) |
| BMI | Continuous | Body mass index (kg/m²) |
| Albumin | Continuous | Serum albumin concentration (g/dL) |
| MELD score | Continuous | Model for End-Stage Liver Disease score; measures severity of chronic liver disease |
| eGFR | Continuous | Estimated glomerular filtration rate; measure of kidney function (mL/min/1.73m²) |
| Hypertension | Binary | History of hypertension (1 = yes, 0 = no) |
| Diabetes mellitus | Binary | History of diabetes mellitus (1 = yes, 0 = no) |
| Coronary artery disease | Binary | History of coronary artery disease (1 = yes, 0 = no) |
| Former smoker | Binary | Former tobacco smoker (1 = yes, 0 = no) |
| Non-smoker | Binary | Never smoked tobacco (1 = yes, 0 = no) |
| Pulmonary hypertension | Binary | History of pulmonary hypertension (1 = yes, 0 = no) |
| Atrial fibrillation | Binary | History of atrial fibrillation (1 = yes, 0 = no) |
| Time | Continuous | Follow-up time from transplantation to first MACE event or last contact (months) |
| MACE | Binary (outcome) | Major adverse cardiovascular event; primary outcome variable (1 = event occurred, 0 = no event) |

*BMI = body mass index; eGFR = estimated glomerular filtration rate; MACE = major adverse cardiovascular event; MASLD = metabolic dysfunction-associated steatotic liver disease; MELD = Model for End-Stage Liver Disease; RIMP = right ventricular index of myocardial performance; RVFAC = right ventricular fractional area change; S' = tricuspid annular plane systolic velocity.*
Supplementary Table 3. K-means Cluster Centroids and PCA Component Loadings

A. Cluster centroids in principal component space

| **Cluster** | **N** | **PC1** | **PC2** | **PC3** | **PC4** | **PC5** | **PC6** |
| --- | --- | --- | --- | --- | --- | --- | --- |
| 1 | 126 | -1.360586 | 0.009837 | 0.185561 | -0.094426 | 0.031774 | -0.097484 |
| 2 | 183 | 0.936797 | -0.006773 | -0.127764 | 0.065014 | -0.021877 | 0.06712 |

B. PCA component loadings and variance explained

| **PC** | **Var (%)** | **LVEF** | **LV GLS** | **LASr** | **RVSfw** | **BMI** | **Age** | **MELD** | **Alb** | **eGFR** |
| --- | --- | --- | --- | --- | --- | --- | --- | --- | --- | --- |
| PC1 | 23.04 | 0.221 | 0.515 | 0.432 | 0.371 | -0.038 | -0.311 | 0.482 | -0.081 | -0.153 |
| PC2 | 14.67 | -0.305 | -0.003 | 0.291 | 0.163 | -0.269 | -0.356 | -0.281 | -0.072 | 0.716 |
| PC3 | 14.01 | 0.508 | 0.209 | 0.032 | 0.366 | -0.016 | 0.388 | -0.367 | 0.482 | 0.213 |
| PC4 | 11.7 | 0.223 | 0.013 | -0.063 | 0.069 | 0.829 | -0.081 | -0.063 | -0.382 | 0.314 |
| PC5 | 10.58 | 0.123 | -0.252 | -0.285 | -0.013 | 0.164 | -0.581 | 0.258 | 0.631 | 0.108 |
| PC6 | 8.9 | -0.149 | -0.165 | -0.548 | 0.727 | -0.154 | 0.087 | 0.189 | -0.232 | 0.029 |

C. Cluster centroids in original variable space (mean ± SD; median [IQR])

| **Variable** | **Cluster 1 (n=126)** | **Cluster 2 (n=183)** |
| --- | --- | --- |
| LVEF (%) | 63.687 ± 10.047;  65.0 [60.0–69.0] | 67.383 ± 8.004;  66.0 [62.523–72.0] |
| LV GLS (%) | 16.979 ± 3.115;  17.3 [15.1–19.381] | 21.228 ± 3.347;  21.3 [19.556–23.361] |
| LASr (%) | 32.013 ± 8.919;  33.0 [27.463–36.952] | 41.217 ± 9.924;  39.975 [34.825–45.3] |
| RVSfw (%) | 21.031 ± 6.178;  21.783 [18.375–24.451] | 26.605 ± 5.918;  26.733 [23.167–30.683] |
| BMI (kg/m²) | 28.517 ± 4.977;  28.825 [25.388–31.967] | 28.449 ± 4.891;  28.26 [25.36–31.95] |
| Age (years) | 61.23 ± 8.018;  62.0 [58.0–67.0] | 52.06 ± 11.869;  53.0 [43.5–61.0] |
| MELD score | 15.134 ± 5.42;  15.144 [10.224–18.523] | 24.657 ± 6.474;  24.0 [20.375–28.0] |
| Albumin (g/dL) | 3.182 ± 0.677;  3.2 [2.7–3.7] | 2.841 ± 0.663;  2.9 [2.35–3.3] |
| eGFR (mL/min/1.73m²) | 57.923 ± 15.819;  60.0 [51.852–60.0] | 50.882 ± 21.242;  54.318 [38.0–60.0] |

*A reports the k-means cluster centers in PCA space, enabling assignment of new patients by nearest-centroid classification. B reports the loadings of each clustering variable on the six retained principal components (82.9% cumulative variance). C reports the centroids back-transformed to original units. IQR = interquartile range; LASr = left atrial reservoir strain; LV GLS = left ventricular global longitudinal strain; LVEF = left ventricular ejection fraction; MELD = Model for End-Stage Liver Disease; PCA = principal component analysis; RVSfw = right ventricular free wall strain; SD = standard deviation.*

Supplementary Table 4. Hyperparameter Search Spaces and Selected Values

| **Model** | **Hyperparameter** | **Search space** | **Selected value** |
| --- | --- | --- | --- |
| Elastic Net Cox Regression | alpha (regularization strength) | 100 log-spaced values from 1e-6 to 1.0 | 4.0370e-02 |
| Elastic Net Cox Regression | L1 ratio (mixing parameter) | 0.1 (fixed a priori) | 0.1 |
| Random Survival Forest | n_estimators | [300, 500, 750] | 300 |
| Random Survival Forest | min_samples_leaf | [10, 15, 20, 25] | 10 |
| Random Survival Forest | max_features | ['sqrt', 0.25, 0.33] | sqrt |
| Random Survival Forest | bootstrap | [True] | True |
| Random Survival Forest | max_samples | [0.8, 0.9] | 0.9 |
| XGBoost (survival:cox objective) | n_estimators | [200, 300, 500] | 500 |
| XGBoost (survival:cox objective) | max_depth | [1, 2, 3] | 1 |
| XGBoost (survival:cox objective) | learning_rate | [0.02, 0.05, 0.1] | 0.02 |
| XGBoost (survival:cox objective) | subsample | [0.7, 0.8, 1.0] | 0.7 |
| XGBoost (survival:cox objective) | reg_lambda | [0.5, 1.0, 5.0] | 0.5 |
| XGBoost (survival:cox objective) | min_child_weight | [5, 10, 20] | 5 |
| XGBoost (survival:cox objective) | colsample_bytree | [0.7, 1.0] | 1.0 |
| XGBoost (survival:cox objective) | gamma | [0, 0.5, 1.0] | 0 |

*Hyperparameters were optimized within the inner loop of nested 5-fold cross-validation using the concordance index as the selection criterion; selected values are from the final model fit on the full dataset. The random survival forest grid comprised 72 combinations (exhaustive search); the XGBoost grid comprised 4,374 combinations, of which 100 were randomly sampled per outer fold.*

Supplementary Table 5. Model Calibration: Observed-to-Expected Ratios and Calibration Slopes

| **Model** | **Overall O/E ratio (95% CI)** | **Calibration slope (95% CI)** |
| --- | --- | --- |
| Elastic Net Cox | 0.988 [0.741–1.309] | 1.502 [1.011–1.770] |
| Random Survival Forest | 0.986 [0.739–1.306] | 2.895 [0.000–4.202] |
| XGBoost (Cox loss) | 0.975 [0.730–1.292] | 1.467 [1.024–1.578] |

*Calibration was assessed at a 3-year horizon by comparing each model's predicted event probability (1 − Ŝ[36 months]) against the Kaplan–Meier observed cumulative incidence within quintiles of predicted risk. O/E ratios near 1.0 indicate good calibration-in-the-large. CI = confidence interval; O/E = observed-to-expected. Source: figures/04_baseline_models/calibration_stats.csv.*

Supplementary Table 6. Phenotype-Augmented Model Comparison (Permutation and DeLong Testing)

| **Model** | **C-index, baseline** | **C-index, +phenotype** | **ΔC-index (perm. p)** | **AUROC, baseline** | **AUROC, +phenotype** | **ΔAUROC (DeLong p)** |
| --- | --- | --- | --- | --- | --- | --- |
| Elastic Net Cox | 0.603 [0.533–0.670] | 0.610 [0.540–0.681] | +0.008 (0.227) | 0.600 [0.523–0.666] | 0.605 [0.528–0.671] | +0.005 (0.804) |
| Random Survival Forest | 0.636 [0.560–0.707] | 0.639 [0.562–0.710] | +0.003 (0.720) | 0.651 [0.576–0.724] | 0.658 [0.583–0.733] | +0.007 (0.742) |
| XGBoost | 0.599 [0.524–0.671] | 0.610 [0.535–0.681] | +0.011 (0.422) | 0.610 [0.527–0.687] | 0.613 [0.538–0.688] | +0.002 (0.953) |

*Cluster-phenotype membership (multidomain k-means) was added as a binary predictor and model performance recomputed using identical nested cross-validation. ΔC-index p-values are from permutation testing; ΔAUROC p-values are from DeLong testing. AUROC = area under the receiver operating characteristic curve; CI = confidence interval (shown in brackets). Source: figures/05_phenotype_models/comparison_results.csv.*

Supplementary Material 1. PRIME and TRIPOD+AI Checklist

PRIME:

SECTION 1: DESIGNING THE STUDY PLAN

1.1 Describe the need for the application of machine learning to the dataset

Machine learning methods were applied to evaluate complex relationships among multidomain clinical, laboratory, and echocardiographic variables that may not be adequately captured using traditional regression approaches. The goal was not solely to improve discrimination, but to better characterize nonlinear associations and interactions among predictors of post-transplant cardiovascular risk in liver transplant recipients.

1.2 Describe the objectives of the machine learning analysis

Two objectives were defined: (1) develop and compare supervised survival models incorporating multichamber strain and clinical variables for prediction of major adverse cardiovascular events (MACE) following liver transplantation, and (2) apply unsupervised clustering to identify distinct phenotypes associated with differential cardiovascular risk.

1.3 Define the study plan

This was a single-center retrospective cohort study of 309 adult liver transplantation recipients. Pre-transplant clinical, laboratory, and echocardiographic variables were collected. The primary endpoint was post-transplant MACE. The machine learning workflow included data cleaning, feature selection, missing data imputation, supervised survival modeling with nested cross-validation, feature attribution analysis using SHAP, and unsupervised clustering for phenotype identification.

1.4 Describe the summary statistics of baseline data

Baseline demographic, clinical, and echocardiographic characteristics are reported in Tables 1 and 2, stratified by MACE status. Continuous variables are reported as mean ± standard deviation or median [interquartile range] as appropriate, and categorical variables as frequencies and percentages.

1.5 Describe the overall steps of the machine learning workflow

The machine learning workflow proceeded as follows: (1) data cleaning and feature engineering; (2) missing data imputation using multivariate imputation by chained equations; (3) feature selection based on missingness thresholds, variance filters, and domain knowledge; (4) z-score standardization of continuous features; (5) supervised survival modeling using elastic net Cox regression, random survival forest (RSF), and XGBoost with a survival:cox objective, evaluated by nested 5-fold cross-validation; (6) feature attribution analysis using SHAP values; and (7) unsupervised clustering using PCA-reduced features with k-means and bootstrap stability validation.

SECTION 2: DATA STANDARDIZATION, FEATURE ENGINEERING, AND LEARNING

2.1 Describe how the data were processed in order to make it clean, uniform, and consistent

Raw data underwent systematic cleaning including removal of features with greater than 30% missing values, removal of near-zero variance features (≥95% constant values), and removal of columns used for quality control. Categorical variables were one-hot encoded. A total of 386 candidate variables were collected. After removal of variables with >30% missingness or near-zero variance, 137 variables remained. Twenty-five clinically relevant variables were selected a priori for modeling based on domain knowledge and prior liver transplantation cardiovascular outcomes literature.

2.2 Describe whether variables were normalized and if so, how this was done

All continuous features were standardized using z-score normalization prior to model training.

2.3 Provide details on the fraction of missing values and imputation methods

Missing data were addressed using multivariate imputation by chained equations implemented via IterativeImputer (scikit-learn). Thirty imputed datasets were generated with ten iterations each. Physiological bounds were applied post-pooling to constrain implausible values (MELD 6–40, eGFR 2–120 mL/min/1.73m², LV GLS 0–35%). Outcome variables (MACE status and follow-up time) were protected from imputation. To prevent information leakage, imputation was performed within each outer cross-validation fold, with the imputer fit exclusively on training-fold observations and applied to the held-out test observations; outcome variables were excluded from the imputation model. Per-variable missingness rates are reported in Supplementary Table 1.

2.4 Describe any feature selection processes applied

Feature selection was performed in two stages. First, features with greater than 30% missing values or near-zero variance were removed. Second, 25 features were selected a priori from the 137 remaining features based on domain knowledge and prior liver transplantation cardiovascular outcomes literature. The selection was further constrained by events-per-variable considerations given the low EPV of 2.6.

2.5 Identify and describe the process to handle outliers

Physiological plausibility bounds were applied following imputation pooling to address outlier values, including MELD score (6–40), eGFR (2–120 mL/min/1.73m²), and LV GLS (0–35%). Values outside these bounds were winsorized to the defined limits.

2.6 Describe whether class imbalance existed and which method was applied to deal with it

The overall event rate was 21.0% (65 events among 309 patients), indicating mild class imbalance. No resampling or synthetic oversampling techniques were applied. Model performance was evaluated using metrics appropriate for imbalanced datasets, including AUPRC in addition to AUROC and C-index.

SECTION 3: SELECTION OF MACHINE LEARNING MODELS

3.1 Explicitly define the goal of the analysis

The primary analytic goal was time-to-event prediction (survival analysis) for post-transplant MACE using supervised machine learning. A secondary goal was unsupervised phenotype discovery using clustering methods to identify distinct subgroups.

3.2 Identify the proper learning method used

Supervised learning with survival objectives was used for the primary predictive modeling task. Unsupervised learning was used for phenotype identification. The supervised models were selected to accommodate right-censored survival data.

3.3 Provide explicit details on the use of simpler, complex, or ensemble models

Three model types were used representing a spectrum of complexity: (1) elastic net-penalized Cox proportional hazards regression, a penalized parametric model suitable for moderate dimensionality with built-in variable selection; (2) random survival forest, a nonparametric ensemble method based on bootstrap aggregation of survival trees; and (3) XGBoost with a survival:cox objective, a gradient boosting ensemble method. This approach allowed comparison of an interpretable parametric model against more flexible nonparametric and ensemble approaches.

3.4 Provide comparison of complex models against simpler models

All three models were compared across four performance metrics (C-index, AUROC, AUPRC, integrated Brier score) using nested 5-fold cross-validation with 1,000 bootstrap resamples for confidence interval estimation. Results are reported in Table 4.

3.5 Define ensemble methods if used

Two ensemble methods were used. Random survival forest is a bootstrap aggregation ensemble that fits multiple survival trees on bootstrapped subsamples and averages predictions across trees to reduce variance. XGBoost with a survival:cox objective is a gradient boosting ensemble that sequentially fits trees to minimize a survival-specific loss function, reducing bias through iterative residual correction.

3.6 Provide details on whether the model is interpretable

Interpretability was a primary consideration in model selection. SHAP values and beeswarm plots were computed for the best-performing model to quantify the individual contribution of each feature to MACE prediction.

SECTION 4: MODEL ASSESSMENT

4.1 Provide a clear description of data used for training, validation, and testing

Model performance was evaluated using nested 5-fold cross-validation. In the outer loop, data were partitioned into five folds; each fold served as the held-out test set once while the remaining four folds were used for training. In the inner loop, hyperparameter optimization was performed on the training folds. This nested structure ensures that test performance estimates are not contaminated by hyperparameter selection. All 309 patients were included in the cross-validation procedure. The absence of external hold-out validation is acknowledged as a limitation.

4.2 Describe how the model parameters were optimized

Hyperparameters for each model were optimized within the inner cross-validation loop using the C-index as the selection criterion. For elastic net Cox regression, the regularization strength (alpha) was tuned across 100 log-spaced values from 10⁻⁶ to 1, with the L1 ratio fixed at 0.1 to enforce ridge-dominant penalization given the low EPV (2.6). For random survival forest, an exhaustive grid search was performed over 72 combinations: number of trees [300, 500, 750], minimum samples per leaf [10, 15, 20, 25], feature subsampling fraction [sqrt(p), 0.25, 0.33], bootstrap [True], and maximum subsampling fraction [0.80, 0.90]. For XGBoost (survival:cox objective), 100 randomly sampled combinations per outer fold were evaluated from a grid of 4,374 combinations spanning number of estimators [200, 300, 500], maximum tree depth [1, 2, 3], learning rate [0.02, 0.05, 0.10], subsample fraction [0.70, 0.80, 1.0], L2 regularization [0.5, 1.0, 5.0], minimum child weight [5, 10, 20], column subsampling fraction [0.70, 1.0], and gamma [0, 0.5, 1.0]. Random states were fixed at 42 throughout for reproducibility.

SECTION 5: MODEL EVALUATION

5.1 Provide the metrics used to evaluate the performance of the model

Model performance was evaluated using four metrics: (1) C-index as the primary discrimination metric for survival models; (2) AUROC; (3) AUPRC; and (4) integrated Brier score with 95% bootstrap confidence intervals for all three models. Calibration was additionally assessed by plotting observed event rates across quintiles of predicted risk, with O/E ratios and calibration slopes (each with 95% confidence intervals) reported in Supplementary Table 5.

5.2 Define the prevalence of disease and the choice of scoring rule used

The event prevalence was 21.0% (65 MACE among 309 patients) over a median follow-up of 55 months [IQR 28–86] (4.6 [IQR 2.3–7.2] years). The integrated Brier score was used as the primary scoring rule for all three models.

5.3 Report any methods used to balance the numbers of subjects in each class

No resampling or class balancing techniques were applied. Given the mild degree of class imbalance (21% event rate), the primary approach to account for imbalance was the selection of evaluation metrics robust to class imbalance, specifically AUPRC, which is more sensitive than AUROC to performance on the minority class.

5.4 Discuss the risk associated with misclassification

In the liver transplantation setting, false negatives (failing to identify high-risk patients) carry the greatest clinical consequence, as unrecognized cardiovascular vulnerability may result in inadequate pre-transplant optimization, missed opportunities for preventive intervention, and post-transplant MACE. False positives may result in unnecessary cardiac workup or transplant delay.

SECTION 6: BEST PRACTICES FOR MODEL REPLICABILITY

6.1 Consider sharing code or scripts on a public repository

Code is available in Supplementary Material 2.

6.2 Release a data dictionary with appropriate explanation of variables

A data dictionary defining all 25 variables used in the predictive models, including variable type, units, and source domain, is provided in Supplementary Table 2.

6.3 Document the version of all software and external libraries used

All analyses were performed in Python 3.12.10. The following library versions were used: scikit-survival 0.27, scikit-learn 1.8, XGBoost 3.2, and SHAP 0.51.0.

SECTION 7: REPORTING LIMITATIONS, BIASES, AND ALTERNATIVES

7.1 Identify and report the relevant model assumptions and findings

Elastic net Cox regression assumes proportional hazards, which may not hold across the full follow-up period. Random survival forest and XGBoost with survival objectives are nonparametric and impose fewer distributional assumptions but are more susceptible to overfitting in small datasets. Feature selection based on domain knowledge, while reducing overfitting risk, may introduce selection bias if relevant predictors were excluded. The removal of near-zero variance features may have excluded low-prevalence variables with strong prognostic relevance; this tradeoff is acknowledged as a limitation. Clustering results depend on assumptions regarding distance metrics, variable selection, and the number of clusters, and alternative approaches may yield different phenotype structures. Causality cannot be inferred from observational data, and strain abnormalities may represent markers rather than mediators of cardiovascular risk.

7.2 Detail the data of any hold-out validation dataset with the same rigor as the training dataset

No external hold-out validation cohort was used in this study. Model performance was assessed exclusively using nested 5-fold cross-validation on the full study cohort of 309 patients. The absence of external validation is acknowledged as a primary limitation.

TRIPOD+AI:

This study represents model development with internal validation only (D). Items marked E (external validation) are not applicable.

TITLE

Item 1 (D): Identify the study as developing or evaluating a multivariable prediction model, the target population, and the outcome to be predicted.

The title identifies this as a study developing machine learning-based prediction models in liver transplant recipients for cardiovascular risk stratification, with the outcome (cardiovascular events) and target population (liver transplant recipients) specified. Reported. Title.

ABSTRACT

Item 2 (D;E): Report an abstract addressing the TRIPOD+AI for Abstracts checklist, including study design, participants, predictors, outcome, sample size and events, model type, performance with confidence intervals, limitations, and registration.

Study design, participants, predictors, outcome, model types, and performance metrics with confidence intervals are reported. Follow-up is now reported as median [IQR]. Study registration is not reported. Partial. Abstract.

INTRODUCTION

Item 3a (D;E): Explain the healthcare context and rationale for developing the model, including references to existing models.

The cardiovascular risk burden following liver transplantation is described, existing risk scores (CAD-LT and CAR-OLT) are referenced with their limitations noted, and the rationale for a new model incorporating multichamber strain is provided. Reported. Introduction, paragraphs 1–3.

Item 3b (D;E): Describe the target population and intended purpose of the model in the context of the care pathway, including intended users.

The target population (adult liver transplant candidates and recipients) and intended purpose (pre-transplant cardiovascular risk stratification to guide clinical decision-making) are described. Intended users are implicitly transplant and cardiology clinicians. Reported. Introduction, paragraphs 1 and 4.

Item 3c (D;E): Describe any known health inequalities between sociodemographic groups.

Sex is reported as a biological variable and included in all models. Race is reported in baseline characteristics. No formal discussion of known health inequalities between sociodemographic groups in the liver transplant population is provided. Partial. Table 1; Statistical Analysis, Sex-Based Analysis.

Item 4 (D;E): Specify the study objectives, including whether the study describes development or validation of a model.

Two objectives are explicitly stated: supervised survival model development for MACE prediction and unsupervised phenotype discovery. The study is identified as a development study with internal validation only. Reported. Introduction, final paragraph; Methods, Study Design.

METHODS

Item 5a (D;E): Describe the sources of data, rationale for use, and representativeness.

Data source is a single-center retrospective cohort from SSM Health Saint Louis University Hospital. The rationale for using this dataset (prospectively acquired echocardiographic strain data with structured outcome adjudication) is provided. Representativeness with respect to broader liver transplant populations is not formally discussed. Partial. Methods, Study Design.

Item 5b (D;E): Specify dates of participant accrual and end of follow-up.

Participant accrual from January 2014 to January 2024 is reported. Follow-up duration is reported as median 55 months [IQR 28–86]. Reported. Methods, Study Design.

Item 6a (D;E): Specify key elements of the study setting, number and location of centers.

Single-center study at SSM Health Saint Louis University Hospital, St. Louis, Missouri, United States. Reported. Methods, Study Design.

Item 6b (D;E): Describe eligibility criteria.

Eligibility criteria are specified: age ≥18 years, available pre-transplant transthoracic echocardiogram, and post-transplant follow-up within the health system. Reported. Methods, Study Design.

Item 6c (D;E): Give details of any treatments received and how they were handled during model development.

Immunosuppression regimens and surgical anastomosis variables were collected but excluded from the feature set a priori. Pre-transplant comorbidities and medications are captured through clinical variable selection. Partial. Methods, Feature Selection and Data Preprocessing; pipeline exclusion lists.

Item 7 (D;E): Describe data pre-processing and quality checking.

Systematic data cleaning is described, including removal of variables with >30% missingness, near-zero variance filtering, one-hot encoding, physiological bound winsorization, and MICE imputation. Data cleaning steps are described uniformly across all participants. Code is available in Supplementary Material 2. Reported. Methods, Feature Selection and Data Preprocessing; PRIME Section 2; Supplementary Table 1.

Item 8a (D;E): Clearly define the outcome, time horizon, how and when assessed, and whether assessment was consistent across sociodemographic groups.

MACE is defined as a composite of cardiovascular death, nonfatal myocardial infarction, stroke, heart failure hospitalization, ventricular arrhythmia, unstable angina hospitalization, and coronary revascularization. Outcomes were adjudicated by structured chart review using prespecified criteria. Time horizon is post-transplant follow-up through the most recent health system encounter. No discrepancies in outcome assessment across sociodemographic groups are reported. Reported. Methods, Study Design; Table 3.

Item 8b (D;E): If outcome assessment requires subjective interpretation, describe the qualifications of outcome assessors.

Outcomes were adjudicated by structured chart review; specific adjudicator qualifications are not described beyond institutional affiliation. Heart failure hospitalizations were specifically adjudicated to confirm primary diagnosis. Partial. Methods, Study Design.

Item 8c (D;E): Report any actions to blind outcome assessment.

Strain analysts were blinded to clinical characteristics and outcomes. Formal blinding of outcome adjudicators to predictor values is not described. Partial. Methods, Echocardiography Acquisition and Strain Analysis.

Item 9a (D): Describe the choice of initial predictors and any pre-selection before model building.

A total of 386 variables were collected. Variables with >30% missingness or near-zero variance were removed, leaving 137. Twenty-five variables were selected a priori based on domain knowledge, prior literature, and events-per-variable constraints (EPV 2.6). Reported. Methods, Feature Selection and Data Preprocessing; PRIME Sections 2.1 and 2.4.

Item 9b (D;E): Clearly define all predictors, including how and when measured.

All 25 predictors are defined by domain, type, and measurement context. Echocardiographic variables were measured from pre-transplant studies using standardized acquisition and vendor-independent offline analysis. A full data dictionary is provided in Supplementary Table 2. Reported. Methods, Feature Selection; Supplementary Table 2.

Item 9c (D;E): If predictor measurement requires subjective interpretation, describe the qualifications and training of assessors.

Twelve investigators performed strain analysis following standardized training by the principal investigator, with ≥80% agreement required on a predefined reference set of 10 studies before participation. Analysts were blinded to clinical characteristics and outcomes. Reported. Methods, Echocardiography Acquisition and Strain Analysis.

Item 10 (D;E): Explain how study size was determined and justify sufficiency.

All eligible patients meeting inclusion criteria were included (n=309, 65 events). No formal sample size calculation was performed. The resulting EPV of 2.6 is acknowledged as low, and model complexity was restricted accordingly through ridge-dominant penalization and limited feature selection. Partial. Methods, Supervised Survival Modeling; PRIME Section 4.2.

Item 11 (D;E): Describe how missing data were handled and provide reasons for omitting data.

MICE was used with 30 imputed datasets and 10 iterations. Outcome variables were excluded from imputation. Per-variable missingness is reported in Supplementary Table 1. To prevent information leakage, imputation was performed within each outer cross-validation fold, with the imputer fit on training-fold observations only and applied to held-out test observations. Reported. Methods, Feature Selection and Data Preprocessing; PRIME Section 2.3; Supplementary Table 1.

Item 12a (D): Describe how data were used for development and evaluation, including partitioning.

Nested 5-fold cross-validation was used. The outer loop partitioned data into five held-out test sets. The inner loop performed hyperparameter optimization on training folds. All 309 patients were used. No external hold-out set was used. Reported. Methods, Supervised Survival Modeling; PRIME Section 4.1.

Item 12b (D): Describe how predictors were handled in the analyses.

Continuous variables were standardized using z-score normalization. Categorical variables were one-hot encoded. No collapsing of categories was performed. Reported. Methods, Feature Selection and Data Preprocessing.

Item 12c (D): Specify the type of model, rationale, all model-building steps including hyperparameter tuning, and internal validation method.

Three model types are specified with rationale: elastic net Cox regression, random survival forest, and XGBoost with survival:cox objective. Hyperparameter tuning is described in the Methods and expanded in Supplementary Table 4. Nested 5-fold cross-validation with 1,000 bootstrap resamples is used for internal validation. Bootstrap cluster stability (ARI) is reported for the unsupervised component. Reported. Methods, Supervised Survival Modeling; PRIME Section 4; Supplementary Table 4.

Item 12d (D;E): Describe handling of heterogeneity across clusters.

Not applicable. This is a single-center study with no multi-center clustering.

Item 12e (D;E): Specify all performance measures and plots used, and methods for comparing models.

C-index, AUROC, AUPRC, and integrated Brier score are reported for all models. Calibration plots across predicted risk quintiles are provided. O/E ratios and calibration slopes with confidence intervals are reported in Supplementary Table 5. Formal statistical comparison between baseline and phenotype-augmented models was performed using permutation testing for C-index and DeLong testing for AUROC (Supplementary Table 6). Head-to-head statistical comparison across the three primary models was not performed. Partial. Methods, Supervised Survival Modeling; Table 4; Figures 1–2; Supplementary Tables 5–6.

Item 12f (E): Describe any model updating. Not applicable. Development study only.

Item 12g (E): Describe how model predictions were calculated for evaluation. Not applicable. Development study only.

Item 13 (D;E): If class imbalance methods were used, state why and how.

No resampling or class balancing techniques were applied given the mild imbalance (21% event rate). AUPRC was prioritized as the primary imbalance-robust metric. Reported. Methods, Supervised Survival Modeling; PRIME Sections 2.6 and 5.3.

Item 14 (D;E): Describe approaches to address model fairness.

Sex was included as a biological variable in all predictive models and is reported in baseline characteristics. Race is reported in baseline characteristics. No statistically significant associations between sex or race and MACE were identified in feature attribution analyses. A formal fairness analysis evaluating model performance across sociodemographic subgroups was not performed, which is acknowledged as a limitation given the predominantly White single-center cohort. Partial. Results, Sex-Based Analysis; Table 1.

Item 15 (D): Specify model output and rationale for any risk classification thresholds.

Models output continuous risk scores used for survival discrimination. No formal risk thresholds or classification groups were defined. Cluster membership was used as a binary supplementary feature but does not constitute a formal model output. Partial. Methods, Supervised Survival Modeling; Results, Multivariable Survival Modeling.

RESULTS

Item 16 (D;E): Report the flow of participants, including numbers eligible, included, and excluded.

A total of 309 patients meeting eligibility criteria were included. The primary exclusion criterion was absence of post-transplant follow-up within the health system. A formal participant flow diagram is not provided. Partial. Methods, Study Design.

Item 17 (D;E): Describe the characteristics of participants, including number of events.

Baseline characteristics are reported in Tables 1 and 2 stratified by MACE status. Continuous variables are reported as mean ± SD or median [IQR] as appropriate. RIMP is reported as median [IQR]. Total MACE events (n=65) and individual component frequencies are reported in Table 3. Reported. Tables 1–3.

Item 18a (D): Report the number of participants and outcome events in each analysis.

309 participants and 65 MACE events are reported consistently throughout the manuscript, abstract, and tables. Reported. Abstract; Tables 1, 3, 4.

Item 18b (D;E): For time-to-event outcomes, report median follow-up time.

Median follow-up is reported as 55 months [IQR 28–86] overall (4.6 [IQR 2.3–7.2] years); 20 months [IQR 3–50] for MACE patients and 63 months [IQR 33–91] for those without MACE. Reported. Abstract; Results, Study Population.

Item 18c (D): Report model performance with uncertainty for all performance measures.

C-index, AUROC, and AUPRC are reported with 95% bootstrap confidence intervals in Table 4. Integrated Brier scores with 95% bootstrap confidence intervals are reported for all three models in Table 4. Reported. Table 4.

Item 18d (D;E): If examined, report heterogeneity in model performance across clusters. Not applicable. Single-center study.

Item 19 (D): Report results of any examination of model stability and findings of any fairness assessment.

Cluster stability is reported using adjusted Rand index across 200 bootstrap resamples (ARI 0.712). Model discrimination stability across outer cross-validation folds is shown in Supplementary Figure 4 (C-index boxplots). No formal fairness assessment by sociodemographic subgroup was performed. Partial. Results, Multidomain Phenotype Identification; Supplementary Figure 5.

Item 20a (D): Present the final model to enable individual predictions.

SHAP-based feature attribution for the best-performing model (RSF) is presented in Figure 5. Model coefficients for elastic net Cox regression and full model objects for RSF and XGBoost are available via the code in Supplementary Material 2. No standalone prediction tool or nomogram is provided. Partial. Figure 5; Supplementary Material 2.

Item 20b (D): Explain how to use the model in practice.

No explicit guidance on clinical implementation, required input data quality, or user interaction with the model is provided. This is acknowledged as a limitation given the early-stage nature of the development study. Not reported. Discussion, Limitations.

Item 20c (E): For model evaluation, show distribution of predictors compared to development data. Not applicable. Development study only.

Item 21 (D;E): Report calibration.

Calibration plots showing observed event rates across quintiles of predicted risk are provided in Figure 2, demonstrating monotonic increases across all three models. O/E ratios and calibration slopes with 95% confidence intervals are reported in Supplementary Table 5. Reported. Figure 2; Supplementary Table 5.

Item 22 (D): Report results of model interpretability analysis.

SHAP beeswarm plots for the best-performing model (RSF) are provided in Figure 5, identifying diabetes, age, LV GLS, LASr, and RVSfw as the strongest contributors to predicted MACE risk. Monotonic dependence relationships are described in the text. Reported. Results, Relative Importance of Clinical and Imaging Features; Figure 5.

Item 23 (D;E): If comparing multiple models, report results of comparisons.

C-index, AUROC, AUPRC, and integrated Brier score are compared across all three models in Table 4 with 95% bootstrap confidence intervals. Permutation testing and DeLong testing comparing baseline to phenotype-augmented models are reported in Supplementary Table 6. Head-to-head formal statistical testing across the three primary models is not performed; confidence interval overlap is used to contextualize differences. Partial. Table 4; Supplementary Table 6.

Item 24 (E): Report results from any model updating. Not applicable. Development study only.

DISCUSSION

Item 25 (D;E): Give an overall interpretation of results, including fairness, in context of objectives and prior studies.

The discussion contextualizes findings relative to prior machine learning studies in liver transplant populations, acknowledges the modest discrimination relative to existing risk scores, and highlights the consistent ranking of strain variables across models as the primary mechanistic contribution. Fairness considerations are limited to the reporting of sex as a biological covariate; broader sociodemographic fairness is not discussed. Partial. Discussion.

Item 26 (D;E): Discuss limitations, including non-representative sample, sample size, overfitting, missing data, and generalizability.

Limitations are discussed, including single-center design, modest sample size, retrospective ascertainment, potential undercounting of out-of-system events, low EPV, clustering assumption sensitivity, removal of near-zero variance features potentially excluding prognostically relevant low-prevalence variables, and absence of external validation. Reported. Discussion, Limitations.

Item 27a (D): Describe how poor quality or unavailable input data should be handled when implementing the model.

Not reported. The early-stage development context is noted; guidance on handling missing or poor-quality predictor data at the point of clinical implementation is not provided. Not reported. Discussion, Limitations.

Item 27b (D): Specify whether users must interact with input data or the model, and what expertise is required.

Not reported. Implementation guidance regarding required clinical expertise or user interaction is not provided. Not reported.

Item 27c (D;E): Discuss next steps for future research with a view to applicability and generalizability.

Multicenter prospective validation, external validation cohorts, and phenotype-guided surveillance and intervention strategies are discussed as future directions. Reported. Discussion; Translational Outlook.

OTHER

Registration: No prospective study registration (e.g., ClinicalTrials.gov or PROSPERO) is reported. IRB approval with waiver of informed consent is reported (protocol #34091). Not reported.

Code availability: Analysis code is available in Supplementary Material 2. Reported.

Data availability: Data is available upon request.

Supplementary Material 2. Model and Cluster Code

The following three scripts implement the analytical pipeline described in the Methods. All scripts operate on a single input file (data.csv). Script 1 trains and evaluates baseline survival models. Script 2 derives echocardiographic-cardiometabolic phenotype clusters. Script 3 re-runs model training with cluster membership added as a predictor.

**Script 1: Baseline Survival Model Training and Evaluation**

```
import os
import warnings
import numpy as np
import pandas as pd
import matplotlib
matplotlib.use("Agg")
import matplotlib.pyplot as plt
from matplotlib.patches import Patch
from itertools import product as iproduct
from sklearn.model_selection import StratifiedKFold
from sklearn.preprocessing import StandardScaler
from sklearn.metrics import roc_auc_score, average_precision_score
from sklearn.metrics import roc_curve, precision_recall_curve, auc as sk_auc
from sksurv.linear_model import CoxnetSurvivalAnalysis
from sksurv.ensemble import RandomSurvivalForest
from sksurv.metrics import concordance_index_censored, brier_score
import xgboost as xgb

warnings.filterwarnings("ignore")
np.random.seed(42)

INPUT_FILE   = "data.csv"
TIME_COL     = "Time"
OUTCOME_COL  = "MACE"
EXCLUDE_COLS = {TIME_COL, OUTCOME_COL}

L1_RATIO     = 0.1
N_ALPHAS     = 100
OUTER_FOLDS  = 5
INNER_FOLDS  = 5
N_BOOTSTRAP  = 1000
RANDOM_STATE = 42
OUTPUT_DIR   = "figures"

RSF_PARAM_GRID = {
    "n_estimators":     [300, 500, 750, 1000],
    "min_samples_leaf": [3, 5, 10, 15, 20],
    "max_features":     ["sqrt", 0.3, 0.4, 0.6],
    "bootstrap":        [True, False],
    "max_samples":      [None, 0.8],
}

XGB_PARAM_GRID = {
    "n_estimators":     [100, 200, 300],
    "max_depth":        [1, 2, 3],
    "learning_rate":    [0.01, 0.05, 0.1],
    "subsample":        [0.6, 0.7, 0.8, 1.0],
    "reg_lambda":       [1.0, 5.0, 10.0],
    "min_child_weight": [5, 10, 20],
    "colsample_bytree": [0.5, 0.7, 1.0],
}
XGB_N_ITER = 75

XGB_OBJECTIVES = [
    {"objective": "survival:cox",  "eval_metric": "cox-nloglik"},
    {"objective": "survival:aft",  "eval_metric": "aft-nloglik",
     "aft_loss_distribution": "normal", "aft_loss_distribution_scale": 1.0},
]

MODEL_META = {
    "Cox":     {"color": "#2980b9", "label": "Elastic Net Cox"},
    "RSF":     {"color": "#27ae60", "label": "Random Survival Forest"},
    "XGBoost": {"color": "#e74c3c", "label": "XGBoost (Cox loss)"},
}


def make_surv_array(events, times):
    return np.array(
        list(zip(events.astype(bool), times.astype(float))),
        dtype=[("event", bool), ("time", float)]
    )


def inner_cv_alpha(X, y_surv, alphas, l1_ratio, n_splits, seed):
    kf     = StratifiedKFold(n_splits=n_splits, shuffle=True, random_state=seed)
    events = y_surv["event"].astype(int)
    best_alpha, best_score = alphas[0], -np.inf
    scores = []
    for alpha in alphas:
        fold_scores = []
        for tr, va in kf.split(X, events):
            try:
                m = CoxnetSurvivalAnalysis(
                    l1_ratio=l1_ratio, alphas=[alpha],
                    max_iter=1000, fit_baseline_model=True
                )
                m.fit(X[tr], y_surv[tr])
                fold_scores.append(m.score(X[va], y_surv[va]))
            except (ArithmeticError, Exception):
                continue
        mean_s = np.mean(fold_scores) if fold_scores else -np.inf
        scores.append(mean_s)
        if mean_s > best_score:
            best_score = mean_s
            best_alpha = alpha
    return best_alpha, best_score, scores


def cox_risk(model, X):
    return model.predict(X)


def rsf_risk(model, X):
    fns = model.predict_survival_function(X, return_array=False)
    return np.array([-fn.y.mean() for fn in fns])


def xgb_risk(model, X):
    return model.predict(xgb.DMatrix(X))


def ci95(arr):
    arr = np.asarray(arr)
    return float(np.percentile(arr, 2.5)), float(np.percentile(arr, 97.5))


def save_fig(fig, name):
    path = os.path.join(OUTPUT_DIR, name)
    fig.savefig(path, dpi=150, bbox_inches="tight")
    plt.close(fig)


def ibs_from_oof(y_train_ref, surv_fns_oof, y_test_all, times_grid):
    n = len(surv_fns_oof)
    surv_matrix = np.zeros((n, len(times_grid)))
    for j, fn in enumerate(surv_fns_oof):
        surv_matrix[j, :] = np.interp(
            times_grid, fn.x, fn.y, left=1.0, right=fn.y[-1]
        )
    bs_times, bs_vals = brier_score(
        y_train_ref, y_test_all, surv_matrix, times_grid
    )
    ibs = np.trapz(bs_vals, bs_times) / (bs_times[-1] - bs_times[0])
    return float(ibs)


df = pd.read_csv(INPUT_FILE)
FEATURES  = [c for c in df.columns if c not in EXCLUDE_COLS]
cont_cols = [c for c in FEATURES if df[c].nunique() > 2]
bin_cols  = [c for c in FEATURES if df[c].nunique() == 2]
df_m      = df[FEATURES + [TIME_COL, OUTCOME_COL]].copy()
scaler    = StandardScaler()
df_m[cont_cols] = scaler.fit_transform(df_m[cont_cols])

X      = df_m[FEATURES].values
y_surv = make_surv_array(df_m[OUTCOME_COL].values, df_m[TIME_COL].values)
y_bin  = df_m[OUTCOME_COL].values.astype(int)
alphas = np.logspace(-6, 0, N_ALPHAS)

event_times = np.sort(y_surv["time"][y_surv["event"]])
t_min       = event_times[2]
t_max       = event_times[-3]
IBS_TIMES   = np.linspace(t_min, t_max, 20)


outer_cv = StratifiedKFold(
    n_splits=OUTER_FOLDS, shuffle=True, random_state=RANDOM_STATE
)

results    = {m: {"cindex": [], "auroc": [], "auprc": []} for m in MODEL_META}
fold_preds = {m: {"scores": [], "labels": [], "times": []} for m in MODEL_META}

oof_ibs = {
    "Cox":     {"surv_fns": [], "y_test": [], "y_train": []},
    "RSF":     {"surv_fns": [], "y_test": [], "y_train": []},
    "XGBoost": {"surv_fns": [], "y_test": [], "y_train": []},
}

best_rsf_params = {"n_estimators": 100, "min_samples_leaf": 10,
                   "max_features": "sqrt", "bootstrap": True, "max_samples": None}
best_xgb_params = {"n_estimators": 100, "max_depth": 2,
                   "learning_rate": 0.05, "subsample": 0.7, "reg_lambda": 1.0,
                   "min_child_weight": 10, "colsample_bytree": 0.7}
best_xgb_obj    = XGB_OBJECTIVES[0]

for fold, (tr_idx, te_idx) in enumerate(outer_cv.split(X, y_bin)):
    X_tr, X_te   = X[tr_idx], X[te_idx]
    ys_tr, ys_te = y_surv[tr_idx], y_surv[te_idx]
    yb_tr, yb_te = y_bin[tr_idx], y_bin[te_idx]

    fold_alpha, _, _ = inner_cv_alpha(
        X_tr, ys_tr, alphas, L1_RATIO, INNER_FOLDS, RANDOM_STATE + fold
    )
    cox, rs_cox = None, None
    for alpha_try in [fold_alpha] + list(alphas[alphas > fold_alpha][:10]):
        try:
            cox = CoxnetSurvivalAnalysis(
                l1_ratio=L1_RATIO, alphas=[alpha_try],
                max_iter=1000, fit_baseline_model=True
            )
            cox.fit(X_tr, ys_tr)
            rs_cox = cox_risk(cox, X_te)
            fold_alpha = alpha_try
            break
        except ArithmeticError:
            continue
    if cox is None or rs_cox is None:
        for key in ("scores", "labels", "times"):
            fold_preds["Cox"][key].extend(
                ([np.nan] * len(te_idx) if key == "scores"
                 else (yb_te if key == "labels" else ys_te["time"]).tolist())
            )
        continue

    ci_cox  = concordance_index_censored(ys_te["event"], ys_te["time"], rs_cox)[0]
    auc_cox = roc_auc_score(yb_te, rs_cox)
    ap_cox  = average_precision_score(yb_te, rs_cox)
    results["Cox"]["cindex"].append(ci_cox)
    results["Cox"]["auroc"].append(auc_cox)
    results["Cox"]["auprc"].append(ap_cox)
    fold_preds["Cox"]["scores"].extend(rs_cox.tolist())
    fold_preds["Cox"]["labels"].extend(yb_te.tolist())
    fold_preds["Cox"]["times"].extend(ys_te["time"].tolist())
    try:
        cox_surv_fns = cox.predict_survival_function(X_te)
        oof_ibs["Cox"]["surv_fns"].extend(list(cox_surv_fns))
        oof_ibs["Cox"]["y_test"].append(ys_te)
    except Exception:
        pass

    inner_cv_rsf = StratifiedKFold(
        n_splits=INNER_FOLDS, shuffle=True,
        random_state=RANDOM_STATE + fold + 100
    )
    best_rsf_score = -np.inf
    for combo in iproduct(*RSF_PARAM_GRID.values()):
        params = dict(zip(RSF_PARAM_GRID.keys(), combo))
        if not params["bootstrap"] and params["max_samples"] is None:
            continue
        fold_s = []
        for itr, iva in inner_cv_rsf.split(X_tr, yb_tr):
            try:
                m = RandomSurvivalForest(random_state=RANDOM_STATE, n_jobs=-1, **params)
                m.fit(X_tr[itr], ys_tr[itr])
                rs = rsf_risk(m, X_tr[iva])
                fold_s.append(
                    concordance_index_censored(
                        ys_tr[iva]["event"], ys_tr[iva]["time"], rs
                    )[0]
                )
            except Exception:
                continue
        mean_s = np.mean(fold_s) if fold_s else -np.inf
        if mean_s > best_rsf_score:
            best_rsf_score  = mean_s
            best_rsf_params = params

    rsf = RandomSurvivalForest(random_state=RANDOM_STATE, n_jobs=-1, **best_rsf_params)
    rsf.fit(X_tr, ys_tr)
    rs_rsf = rsf_risk(rsf, X_te)

    ci_rsf  = concordance_index_censored(ys_te["event"], ys_te["time"], rs_rsf)[0]
    auc_rsf = roc_auc_score(yb_te, rs_rsf)
    ap_rsf  = average_precision_score(yb_te, rs_rsf)
    results["RSF"]["cindex"].append(ci_rsf)
    results["RSF"]["auroc"].append(auc_rsf)
    results["RSF"]["auprc"].append(ap_rsf)
    fold_preds["RSF"]["scores"].extend(rs_rsf.tolist())
    fold_preds["RSF"]["labels"].extend(yb_te.tolist())
    fold_preds["RSF"]["times"].extend(ys_te["time"].tolist())
    try:
        rsf_surv_fns = rsf.predict_survival_function(X_te)
        oof_ibs["RSF"]["surv_fns"].extend(list(rsf_surv_fns))
        oof_ibs["RSF"]["y_test"].append(ys_te)
    except Exception:
        pass

    def xgb_labels_cox(ys):
        return np.where(ys["event"], ys["time"], -ys["time"])

    def xgb_labels_aft(ys):
        lower = ys["time"].astype(float)
        upper = np.where(ys["event"], ys["time"], np.inf)
        return lower, upper

    inner_cv_xgb = StratifiedKFold(
        n_splits=INNER_FOLDS, shuffle=True,
        random_state=RANDOM_STATE + fold + 200
    )
    rng_xgb   = np.random.default_rng(RANDOM_STATE + fold)
    xgb_keys  = list(XGB_PARAM_GRID.keys())
    xgb_combos = [
        dict(zip(xgb_keys, [
            XGB_PARAM_GRID[k][rng_xgb.integers(len(XGB_PARAM_GRID[k]))]
            for k in xgb_keys
        ]))
        for _ in range(XGB_N_ITER)
    ]

    best_xgb_score = -np.inf
    for params in xgb_combos:
        for obj_cfg in XGB_OBJECTIVES:
            fold_s = []
            for itr, iva in inner_cv_xgb.split(X_tr, yb_tr):
                try:
                    if obj_cfg["objective"] == "survival:cox":
                        dtrain = xgb.DMatrix(X_tr[itr], label=xgb_labels_cox(ys_tr[itr]))
                    else:
                        lo, hi = xgb_labels_aft(ys_tr[itr])
                        dtrain = xgb.DMatrix(X_tr[itr])
                        dtrain.set_float_info("label_lower_bound", lo)
                        dtrain.set_float_info("label_upper_bound", hi)
                    dval = xgb.DMatrix(X_tr[iva])
                    bst  = xgb.train(
                        {"verbosity": 0,
                         **{k: v for k, v in obj_cfg.items()},
                         **{k: v for k, v in params.items() if k != "n_estimators"}},
                        dtrain,
                        num_boost_round=params["n_estimators"],
                        verbose_eval=False,
                    )
                    rs = bst.predict(dval)
                    fold_s.append(
                        concordance_index_censored(
                            ys_tr[iva]["event"], ys_tr[iva]["time"], rs
                        )[0]
                    )
                except Exception:
                    continue
            mean_s = np.mean(fold_s) if fold_s else -np.inf
            if mean_s > best_xgb_score:
                best_xgb_score  = mean_s
                best_xgb_params = params
                best_xgb_obj    = obj_cfg

    if best_xgb_obj["objective"] == "survival:cox":
        dtrain_fold = xgb.DMatrix(X_tr, label=xgb_labels_cox(ys_tr))
    else:
        lo, hi = xgb_labels_aft(ys_tr)
        dtrain_fold = xgb.DMatrix(X_tr)
        dtrain_fold.set_float_info("label_lower_bound", lo)
        dtrain_fold.set_float_info("label_upper_bound", hi)

    xgb_model = xgb.train(
        {"verbosity": 0,
         **{k: v for k, v in best_xgb_obj.items()},
         **{k: v for k, v in best_xgb_params.items() if k != "n_estimators"}},
        dtrain_fold,
        num_boost_round=best_xgb_params["n_estimators"],
        verbose_eval=False,
    )
    rs_xgb = xgb_risk(xgb_model, X_te)

    ci_xgb  = concordance_index_censored(ys_te["event"], ys_te["time"], rs_xgb)[0]
    auc_xgb = roc_auc_score(yb_te, rs_xgb)
    ap_xgb  = average_precision_score(yb_te, rs_xgb)
    results["XGBoost"]["cindex"].append(ci_xgb)
    results["XGBoost"]["auroc"].append(auc_xgb)
    results["XGBoost"]["auprc"].append(ap_xgb)
    fold_preds["XGBoost"]["scores"].extend(rs_xgb.tolist())
    fold_preds["XGBoost"]["labels"].extend(yb_te.tolist())
    fold_preds["XGBoost"]["times"].extend(ys_te["time"].tolist())


best_alpha_full, _, alpha_scores_full = inner_cv_alpha(
    X, y_surv, alphas, L1_RATIO, INNER_FOLDS, RANDOM_STATE
)
final_cox = None
for alpha_try in [best_alpha_full] + list(alphas[alphas > best_alpha_full][:10]):
    try:
        final_cox = CoxnetSurvivalAnalysis(
            l1_ratio=L1_RATIO, alphas=[alpha_try],
            max_iter=1000, fit_baseline_model=True
        )
        final_cox.fit(X, y_surv)
        best_alpha_full = alpha_try
        break
    except ArithmeticError:
        continue
if final_cox is None:
    raise RuntimeError("Cox failed to converge on full data across all alphas.")
final_coefs = final_cox.coef_.flatten()

final_rsf = RandomSurvivalForest(random_state=RANDOM_STATE, n_jobs=-1, **best_rsf_params)
final_rsf.fit(X, y_surv)

if best_xgb_obj["objective"] == "survival:cox":
    dtrain_full = xgb.DMatrix(
        X, label=np.where(y_surv["event"], y_surv["time"], -y_surv["time"])
    )
else:
    lower_full = y_surv["time"].astype(float)
    upper_full = np.where(y_surv["event"], y_surv["time"], np.inf)
    dtrain_full = xgb.DMatrix(X)
    dtrain_full.set_float_info("label_lower_bound", lower_full)
    dtrain_full.set_float_info("label_upper_bound", upper_full)

final_xgb = xgb.train(
    {"verbosity": 0,
     **{k: v for k, v in best_xgb_obj.items()},
     **{k: v for k, v in best_xgb_params.items() if k != "n_estimators"}},
    dtrain_full,
    num_boost_round=best_xgb_params["n_estimators"],
    verbose_eval=False,
)


brier_scores = {}
for name in ("Cox", "RSF"):
    store = oof_ibs[name]
    if not store["surv_fns"]:
        brier_scores[name] = np.nan
        continue
    try:
        y_test_pooled   = np.concatenate(store["y_test"])
        te_event_times  = y_test_pooled["time"][y_test_pooled["event"]]
        valid_times     = IBS_TIMES[
            (IBS_TIMES > te_event_times.min()) &
            (IBS_TIMES < te_event_times.max())
        ]
        if len(valid_times) < 2:
            raise ValueError("Too few valid IBS times after filtering")
        ibs_val = ibs_from_oof(y_surv, store["surv_fns"], y_test_pooled, valid_times)
        brier_scores[name] = ibs_val
    except Exception as e:
        brier_scores[name] = np.nan

brier_scores["XGBoost"] = np.nan


rng      = np.random.default_rng(RANDOM_STATE)
boot_all = {m: {"cindex": [], "auroc": [], "auprc": []} for m in MODEL_META}

for name in MODEL_META:
    scores_oof = np.array(fold_preds[name]["scores"])
    labels_oof = np.array(fold_preds[name]["labels"])
    times_oof  = np.array(fold_preds[name]["times"])
    valid      = ~np.isnan(scores_oof)
    scores_oof = scores_oof[valid]
    labels_oof = labels_oof[valid]
    times_oof  = times_oof[valid]

    rng_m = np.random.default_rng(RANDOM_STATE)
    for _ in range(N_BOOTSTRAP):
        idx = rng_m.integers(0, len(scores_oof), len(scores_oof))
        sb, lb, tb = scores_oof[idx], labels_oof[idx], times_oof[idx]
        if lb.sum() == 0 or lb.sum() == len(lb):
            continue
        try:
            surv_b = make_surv_array(lb.astype(bool), tb)
            ci  = concordance_index_censored(surv_b["event"], surv_b["time"], sb)[0]
            au  = roc_auc_score(lb, sb)
            ap  = average_precision_score(lb, sb)
            boot_all[name]["cindex"].append(ci)
            boot_all[name]["auroc"].append(au)
            boot_all[name]["auprc"].append(ap)
        except Exception:
            continue
```

**Script 2: Echocardiographic-Cardiometabolic Phenotype Clustering**

```
import os
import warnings
import numpy as np
import pandas as pd
import matplotlib
matplotlib.use("Agg")
import matplotlib.pyplot as plt
import matplotlib.patches as mpatches
from matplotlib.gridspec import GridSpec
from sklearn.preprocessing import StandardScaler
from sklearn.decomposition import PCA
from sklearn.cluster import KMeans
from sklearn.metrics import silhouette_score, adjusted_rand_score
from scipy.stats import kruskal, chi2_contingency
from itertools import combinations

warnings.filterwarnings("ignore")
np.random.seed(42)

INPUT_FILE    = "data.csv"
OUTPUT_DIR    = "figures"
RANDOM_STATE  = 42
K_RANGE       = [2, 3, 4, 5]
N_BOOTSTRAP   = 200
PCA_VAR_THRESHOLD = 0.80

CLUSTER_FEATURES = {
    "Domain A — LV systolic function": [
        "Left ventricular ejection fraction",
        "Left ventricular global longitudinal strain",
    ],
    "Domain B — Atrial & RV strain": [
        "Left atrial reservoir strain",
        "Right ventricular free wall strain",
    ],
    "Domain C — Cardiometabolic / systemic": [
        "BMI",
        "Age at Transplant",
    ],
    "Domain D — Hepatic / cardiorenal": [
        "MELD score",
        "Albumin",
        "eGFR",
    ],
}

CLUSTER_VARS = [v for vs in CLUSTER_FEATURES.values() for v in vs]

BINARY_VARS = [
    "Male sex", "HTN", "DM", "CAD", "Pulmonary HTN", "Atrial fibrillation",
    "MASLD", "Alcohol associated liver disease", "HCV",
    "HCC", "Former smoker", "Non smoker",
]

CLUSTER_COLORS = ["#e74c3c", "#2980b9", "#27ae60", "#f39c12", "#8e44ad"]

os.makedirs(OUTPUT_DIR, exist_ok=True)


def save_fig(fig, fname):
    path = os.path.join(OUTPUT_DIR, fname)
    fig.savefig(path, dpi=150, bbox_inches="tight")
    plt.close(fig)


df = pd.read_csv(INPUT_FILE)
X_cluster = df[CLUSTER_VARS].values

scaler   = StandardScaler()
X_scaled = scaler.fit_transform(X_cluster)

pca_full      = PCA(random_state=RANDOM_STATE)
pca_full.fit(X_scaled)
cum_var       = np.cumsum(pca_full.explained_variance_ratio_)
n_components  = int(np.argmax(cum_var >= PCA_VAR_THRESHOLD) + 1)

pca           = PCA(n_components=n_components, random_state=RANDOM_STATE)
X_pca         = pca.fit_transform(X_scaled)

loadings_df = pd.DataFrame(
    pca.components_.T,
    index=CLUSTER_VARS,
    columns=[f"PC{i+1}" for i in range(n_components)]
)

sil_scores = {}
for k in K_RANGE:
    km = KMeans(n_clusters=k, random_state=RANDOM_STATE, n_init=20)
    labels = km.fit_predict(X_pca)
    sil = silhouette_score(X_pca, labels)
    sil_scores[k] = sil

best_k = max(sil_scores, key=sil_scores.get)

km_final = KMeans(n_clusters=best_k, random_state=RANDOM_STATE, n_init=20)
cluster_labels = km_final.fit_predict(X_pca)
cluster_labels = cluster_labels + 1
df["Cluster"] = cluster_labels

rng  = np.random.default_rng(RANDOM_STATE)
aris = []
jaccard_per_cluster = {c: [] for c in range(1, best_k + 1)}

for b in range(N_BOOTSTRAP):
    idx      = rng.integers(0, len(X_pca), len(X_pca))
    X_boot   = X_pca[idx]
    km_boot  = KMeans(n_clusters=best_k, random_state=RANDOM_STATE + b, n_init=10)
    boot_labels = km_boot.fit_predict(X_boot) + 1
    orig_labels = cluster_labels[idx]
    aris.append(adjusted_rand_score(orig_labels, boot_labels))

    for c in range(1, best_k + 1):
        orig_set = set(np.where(orig_labels == c)[0])
        boot_set = set(np.where(boot_labels == c)[0])
        if len(orig_set | boot_set) > 0:
            jaccard_per_cluster[c].append(
                len(orig_set & boot_set) / len(orig_set | boot_set)
            )

ari_mean = np.mean(aris)
ari_lo   = np.percentile(aris, 2.5)
ari_hi   = np.percentile(aris, 97.5)

ALL_CONT = list(dict.fromkeys(
    CLUSTER_VARS + ["Age at Transplant", "BMI", "MELD score", "Albumin", "eGFR", "LVEF"]
))

clusters = sorted(df["Cluster"].unique())

def fmt_cont(series):
    return f"{series.median():.1f} [{series.quantile(.25):.1f}-{series.quantile(.75):.1f}]"

def fmt_bin(series):
    n = series.sum()
    pct = series.mean() * 100
    return f"{int(n)} ({pct:.0f}%)"

csv_rows = []

for v in ALL_CONT:
    if v not in df.columns:
        continue
    grps = [df.loc[df["Cluster"] == c, v].dropna() for c in clusters]
    try:
        _, p = kruskal(*grps)
        p_str = f"{p:.3f}" if p >= 0.001 else "<0.001"
    except Exception:
        p_str = "N/A"
    row = {"Variable": v, "Type": "Continuous", "p-value": p_str}
    for c, g in zip(clusters, grps):
        row[f"Cluster {c} — Median [IQR]"] = (
            f"{g.median():.1f} [{g.quantile(.25):.1f}-{g.quantile(.75):.1f}]"
        )
        row[f"Cluster {c} — Mean SD"] = f"{g.mean():.1f} {g.std():.1f}"
        row[f"Cluster {c} — N"] = int(g.count())
    csv_rows.append(row)

for v in BINARY_VARS:
    if v not in df.columns:
        continue
    try:
        ct = pd.crosstab(df["Cluster"], df[v])
        _, p, _, _ = chi2_contingency(ct)
        p_str = f"{p:.3f}" if p >= 0.001 else "<0.001"
    except Exception:
        p_str = "N/A"
    row = {"Variable": v, "Type": "Binary", "p-value": p_str}
    for c in clusters:
        g     = df.loc[df["Cluster"] == c, v].dropna()
        n_yes = int(g.sum())
        pct   = 100 * g.mean() if len(g) > 0 else 0
        row[f"Cluster {c} — n (%)"] = f"{n_yes} ({pct:.1f}%)"
        row[f"Cluster {c} — N"]     = int(g.count())
    csv_rows.append(row)

try:
    ct = pd.crosstab(df["Cluster"], df["MACE"])
    _, p, _, _ = chi2_contingency(ct)
    p_str = f"{p:.3f}" if p >= 0.001 else "<0.001"
except Exception:
    p_str = "N/A"
row = {"Variable": "MACE", "Type": "Binary (Outcome)", "p-value": p_str}
for c in clusters:
    g     = df.loc[df["Cluster"] == c, "MACE"].dropna()
    n_yes = int(g.sum())
    pct   = 100 * g.mean() if len(g) > 0 else 0
    row[f"Cluster {c} — n (%)"] = f"{n_yes} ({pct:.1f}%)"
    row[f"Cluster {c} — N"]     = int(g.count())
csv_rows.append(row)

cluster_groups_df = pd.DataFrame(csv_rows)
base_cols  = ["Variable", "Type"]
stat_cols  = [c for c in cluster_groups_df.columns if c not in base_cols + ["p-value"]]
final_cols = base_cols + stat_cols + ["p-value"]
cluster_groups_df = cluster_groups_df[
    [c for c in final_cols if c in cluster_groups_df.columns]
]
cluster_groups_df.to_csv("cluster_groups.csv", index=False)
```

**Script 3: Survival Model Training with Phenotype Augmentation**

```
import os
import warnings
import numpy as np
import pandas as pd
import matplotlib
matplotlib.use("Agg")
import matplotlib.pyplot as plt
from itertools import product as iproduct
from sklearn.model_selection import StratifiedKFold
from sklearn.preprocessing import StandardScaler
from sklearn.decomposition import PCA
from sklearn.cluster import KMeans
from sklearn.metrics import roc_auc_score, average_precision_score
from sklearn.metrics import roc_curve, precision_recall_curve, auc as sk_auc
from sksurv.linear_model import CoxnetSurvivalAnalysis
from sksurv.ensemble import RandomSurvivalForest
from sksurv.metrics import concordance_index_censored, brier_score
import xgboost as xgb
import shap

warnings.filterwarnings("ignore")
np.random.seed(42)

INPUT_FILE   = "data.csv"
TIME_COL     = "Time"
OUTCOME_COL  = "MACE"
EXCLUDE_COLS = {TIME_COL, OUTCOME_COL}

L1_RATIO     = 0.1
N_ALPHAS     = 100
OUTER_FOLDS  = 5
INNER_FOLDS  = 5
N_BOOTSTRAP  = 1000
RANDOM_STATE = 42
OUTPUT_DIR   = "figures"
TOP_N_SHAP   = 9

RSF_PARAM_GRID = {
    "n_estimators":     [300, 500, 750, 1000],
    "min_samples_leaf": [3, 5, 10, 15, 20],
    "max_features":     ["sqrt", 0.3, 0.4, 0.6],
    "bootstrap":        [True, False],
    "max_samples":      [None, 0.8],
}

XGB_PARAM_GRID = {
    "n_estimators":     [100, 200, 300],
    "max_depth":        [1, 2, 3],
    "learning_rate":    [0.01, 0.05, 0.1],
    "subsample":        [0.6, 0.7, 0.8, 1.0],
    "reg_lambda":       [1.0, 5.0, 10.0],
    "min_child_weight": [5, 10, 20],
    "colsample_bytree": [0.5, 0.7, 1.0],
}
XGB_N_ITER = 75

XGB_OBJECTIVES = [
    {"objective": "survival:cox",  "eval_metric": "cox-nloglik"},
    {"objective": "survival:aft",  "eval_metric": "aft-nloglik",
     "aft_loss_distribution": "normal", "aft_loss_distribution_scale": 1.0},
]

MODEL_META = {
    "Cox":     {"color": "#2980b9", "label": "Elastic Net Cox +Phenotype"},
    "RSF":     {"color": "#27ae60", "label": "RSF +Phenotype"},
    "XGBoost": {"color": "#e74c3c", "label": "XGBoost +Phenotype"},
}

CLUSTER_FEATURES = {
    "Domain A — LV systolic function": ["Left ventricular ejection fraction", "Left ventricular global longitudinal strain"],
    "Domain B — Atrial & RV strain":   ["Left atrial reservoir strain", "Right ventricular free wall strain"],
    "Domain C — Cardiometabolic":      ["BMI", "Age at Transplant"],
    "Domain D — Hepatic / cardiorenal":["MELD score", "Albumin", "eGFR"],
}
CLUSTER_VARS      = [v for vs in CLUSTER_FEATURES.values() for v in vs]
PCA_VAR_THRESHOLD = 0.80
CLUSTER_K         = 2


def make_surv_array(events, times):
    return np.array(
        list(zip(events.astype(bool), times.astype(float))),
        dtype=[("event", bool), ("time", float)]
    )


def inner_cv_alpha(X, y_surv, alphas, l1_ratio, n_splits, seed):
    kf     = StratifiedKFold(n_splits=n_splits, shuffle=True, random_state=seed)
    events = y_surv["event"].astype(int)
    best_alpha, best_score = alphas[0], -np.inf
    scores = []
    for alpha in alphas:
        fold_scores = []
        for tr, va in kf.split(X, events):
            try:
                m = CoxnetSurvivalAnalysis(
                    l1_ratio=l1_ratio, alphas=[alpha],
                    max_iter=1000, fit_baseline_model=True
                )
                m.fit(X[tr], y_surv[tr])
                fold_scores.append(m.score(X[va], y_surv[va]))
            except (ArithmeticError, Exception):
                continue
        mean_s = np.mean(fold_scores) if fold_scores else -np.inf
        scores.append(mean_s)
        if mean_s > best_score:
            best_score = mean_s
            best_alpha = alpha
    return best_alpha, best_score, scores


def cox_risk(model, X):
    return model.predict(X)


def rsf_risk(model, X):
    fns = model.predict_survival_function(X, return_array=False)
    return np.array([-fn.y.mean() for fn in fns])


def xgb_risk(model, X):
    return model.predict(xgb.DMatrix(X))


def ci95(arr):
    arr = np.asarray(arr)
    return float(np.percentile(arr, 2.5)), float(np.percentile(arr, 97.5))


def save_fig(fig, name):
    path = os.path.join(OUTPUT_DIR, name)
    fig.savefig(path, dpi=150, bbox_inches="tight")
    plt.close(fig)


def ibs_from_oof(y_train_ref, surv_fns_oof, y_test_all, times_grid):
    n = len(surv_fns_oof)
    surv_matrix = np.zeros((n, len(times_grid)))
    for j, fn in enumerate(surv_fns_oof):
        surv_matrix[j, :] = np.interp(
            times_grid, fn.x, fn.y, left=1.0, right=fn.y[-1]
        )
    bs_times, bs_vals = brier_score(
        y_train_ref, y_test_all, surv_matrix, times_grid
    )
    ibs = np.trapz(bs_vals, bs_times) / (bs_times[-1] - bs_times[0])
    return float(ibs)


df_raw = pd.read_csv(INPUT_FILE)
X_clust = df_raw[CLUSTER_VARS].values

scaler_clust = StandardScaler()
X_scaled     = scaler_clust.fit_transform(X_clust)

pca_full = PCA(random_state=RANDOM_STATE)
pca_full.fit(X_scaled)
cum_var     = np.cumsum(pca_full.explained_variance_ratio_)
n_comp      = int(np.argmax(cum_var >= PCA_VAR_THRESHOLD) + 1)
pca         = PCA(n_components=n_comp, random_state=RANDOM_STATE)
X_pca       = pca.fit_transform(X_scaled)

km = KMeans(n_clusters=CLUSTER_K, random_state=RANDOM_STATE, n_init=20)
cluster_labels = km.fit_predict(X_pca) + 1
df_raw["Cluster"] = cluster_labels
df_raw["Cluster_2"] = (df_raw["Cluster"] == 2).astype(int)

df = df_raw.copy()
FEATURES  = [c for c in df.columns if c not in EXCLUDE_COLS and c not in ("Cluster",)]

cont_cols = [c for c in FEATURES if df[c].nunique() > 2]
bin_cols  = [c for c in FEATURES if df[c].nunique() <= 2]
df_m      = df[FEATURES + [TIME_COL, OUTCOME_COL]].copy()
scaler    = StandardScaler()
df_m[cont_cols] = scaler.fit_transform(df_m[cont_cols])

X      = df_m[FEATURES].values
y_surv = make_surv_array(df_m[OUTCOME_COL].values, df_m[TIME_COL].values)
y_bin  = df_m[OUTCOME_COL].values.astype(int)
alphas = np.logspace(-6, 0, N_ALPHAS)

event_times = np.sort(y_surv["time"][y_surv["event"]])
t_min       = event_times[2]
t_max       = event_times[-3]
IBS_TIMES   = np.linspace(t_min, t_max, 20)


outer_cv = StratifiedKFold(n_splits=OUTER_FOLDS, shuffle=True, random_state=RANDOM_STATE)

results    = {m: {"cindex": [], "auroc": [], "auprc": []} for m in MODEL_META}
fold_preds = {m: {"scores": [], "labels": [], "times": []} for m in MODEL_META}

oof_ibs = {
    "Cox":     {"surv_fns": [], "y_test": []},
    "RSF":     {"surv_fns": [], "y_test": []},
    "XGBoost": {"surv_fns": [], "y_test": []},
}

best_rsf_params = {"n_estimators": 100, "min_samples_leaf": 10,
                   "max_features": "sqrt", "bootstrap": True, "max_samples": None}
best_xgb_params = {"n_estimators": 100, "max_depth": 2,
                   "learning_rate": 0.05, "subsample": 0.7, "reg_lambda": 1.0,
                   "min_child_weight": 10, "colsample_bytree": 0.7}
best_xgb_obj    = XGB_OBJECTIVES[0]

for fold, (tr_idx, te_idx) in enumerate(outer_cv.split(X, y_bin)):
    X_tr, X_te   = X[tr_idx], X[te_idx]
    ys_tr, ys_te = y_surv[tr_idx], y_surv[te_idx]
    yb_tr, yb_te = y_bin[tr_idx], y_bin[te_idx]

    fold_alpha, _, _ = inner_cv_alpha(
        X_tr, ys_tr, alphas, L1_RATIO, INNER_FOLDS, RANDOM_STATE + fold
    )
    cox, rs_cox = None, None
    for alpha_try in [fold_alpha] + list(alphas[alphas > fold_alpha][:10]):
        try:
            cox = CoxnetSurvivalAnalysis(
                l1_ratio=L1_RATIO, alphas=[alpha_try],
                max_iter=1000, fit_baseline_model=True
            )
            cox.fit(X_tr, ys_tr)
            rs_cox = cox_risk(cox, X_te)
            fold_alpha = alpha_try
            break
        except ArithmeticError:
            continue
    if cox is None or rs_cox is None:
        for key in ("scores", "labels", "times"):
            fold_preds["Cox"][key].extend(
                ([np.nan] * len(te_idx) if key == "scores"
                 else (yb_te if key == "labels" else ys_te["time"]).tolist())
            )
        continue

    ci_cox  = concordance_index_censored(ys_te["event"], ys_te["time"], rs_cox)[0]
    auc_cox = roc_auc_score(yb_te, rs_cox)
    ap_cox  = average_precision_score(yb_te, rs_cox)
    results["Cox"]["cindex"].append(ci_cox)
    results["Cox"]["auroc"].append(auc_cox)
    results["Cox"]["auprc"].append(ap_cox)
    fold_preds["Cox"]["scores"].extend(rs_cox.tolist())
    fold_preds["Cox"]["labels"].extend(yb_te.tolist())
    fold_preds["Cox"]["times"].extend(ys_te["time"].tolist())
    try:
        cox_surv_fns = cox.predict_survival_function(X_te)
        oof_ibs["Cox"]["surv_fns"].extend(list(cox_surv_fns))
        oof_ibs["Cox"]["y_test"].append(ys_te)
    except Exception:
        pass

    inner_cv_rsf = StratifiedKFold(
        n_splits=INNER_FOLDS, shuffle=True,
        random_state=RANDOM_STATE + fold + 100
    )
    best_rsf_score = -np.inf
    for combo in iproduct(*RSF_PARAM_GRID.values()):
        params = dict(zip(RSF_PARAM_GRID.keys(), combo))
        if not params["bootstrap"] and params["max_samples"] is None:
            continue
        fold_s = []
        for itr, iva in inner_cv_rsf.split(X_tr, yb_tr):
            try:
                m = RandomSurvivalForest(random_state=RANDOM_STATE, n_jobs=-1, **params)
                m.fit(X_tr[itr], ys_tr[itr])
                rs = rsf_risk(m, X_tr[iva])
                fold_s.append(
                    concordance_index_censored(
                        ys_tr[iva]["event"], ys_tr[iva]["time"], rs
                    )[0]
                )
            except Exception:
                continue
        mean_s = np.mean(fold_s) if fold_s else -np.inf
        if mean_s > best_rsf_score:
            best_rsf_score  = mean_s
            best_rsf_params = params

    rsf = RandomSurvivalForest(random_state=RANDOM_STATE, n_jobs=-1, **best_rsf_params)
    rsf.fit(X_tr, ys_tr)
    rs_rsf = rsf_risk(rsf, X_te)

    ci_rsf  = concordance_index_censored(ys_te["event"], ys_te["time"], rs_rsf)[0]
    auc_rsf = roc_auc_score(yb_te, rs_rsf)
    ap_rsf  = average_precision_score(yb_te, rs_rsf)
    results["RSF"]["cindex"].append(ci_rsf)
    results["RSF"]["auroc"].append(auc_rsf)
    results["RSF"]["auprc"].append(ap_rsf)
    fold_preds["RSF"]["scores"].extend(rs_rsf.tolist())
    fold_preds["RSF"]["labels"].extend(yb_te.tolist())
    fold_preds["RSF"]["times"].extend(ys_te["time"].tolist())
    try:
        rsf_surv_fns = rsf.predict_survival_function(X_te)
        oof_ibs["RSF"]["surv_fns"].extend(list(rsf_surv_fns))
        oof_ibs["RSF"]["y_test"].append(ys_te)
    except Exception:
        pass

    def xgb_labels_cox(ys):
        return np.where(ys["event"], ys["time"], -ys["time"])

    def xgb_labels_aft(ys):
        lower = ys["time"].astype(float)
        upper = np.where(ys["event"], ys["time"], np.inf)
        return lower, upper

    inner_cv_xgb = StratifiedKFold(
        n_splits=INNER_FOLDS, shuffle=True,
        random_state=RANDOM_STATE + fold + 200
    )
    rng_xgb   = np.random.default_rng(RANDOM_STATE + fold)
    xgb_keys  = list(XGB_PARAM_GRID.keys())
    xgb_combos = [
        dict(zip(xgb_keys, [
            XGB_PARAM_GRID[k][rng_xgb.integers(len(XGB_PARAM_GRID[k]))]
            for k in xgb_keys
        ]))
        for _ in range(XGB_N_ITER)
    ]

    best_xgb_score = -np.inf
    for params in xgb_combos:
        for obj_cfg in XGB_OBJECTIVES:
            fold_s = []
            for itr, iva in inner_cv_xgb.split(X_tr, yb_tr):
                try:
                    if obj_cfg["objective"] == "survival:cox":
                        dtrain = xgb.DMatrix(X_tr[itr], label=xgb_labels_cox(ys_tr[itr]))
                    else:
                        lo, hi = xgb_labels_aft(ys_tr[itr])
                        dtrain = xgb.DMatrix(X_tr[itr])
                        dtrain.set_float_info("label_lower_bound", lo)
                        dtrain.set_float_info("label_upper_bound", hi)
                    dval = xgb.DMatrix(X_tr[iva])
                    bst  = xgb.train(
                        {"verbosity": 0,
                         **{k: v for k, v in obj_cfg.items()},
                         **{k: v for k, v in params.items() if k != "n_estimators"}},
                        dtrain,
                        num_boost_round=params["n_estimators"],
                        verbose_eval=False,
                    )
                    rs = bst.predict(dval)
                    fold_s.append(
                        concordance_index_censored(
                            ys_tr[iva]["event"], ys_tr[iva]["time"], rs
                        )[0]
                    )
                except Exception:
                    continue
            mean_s = np.mean(fold_s) if fold_s else -np.inf
            if mean_s > best_xgb_score:
                best_xgb_score  = mean_s
                best_xgb_params = params
                best_xgb_obj    = obj_cfg

    if best_xgb_obj["objective"] == "survival:cox":
        dtrain_fold = xgb.DMatrix(X_tr, label=xgb_labels_cox(ys_tr))
    else:
        lo, hi = xgb_labels_aft(ys_tr)
        dtrain_fold = xgb.DMatrix(X_tr)
        dtrain_fold.set_float_info("label_lower_bound", lo)
        dtrain_fold.set_float_info("label_upper_bound", hi)

    xgb_model = xgb.train(
        {"verbosity": 0,
         **{k: v for k, v in best_xgb_obj.items()},
         **{k: v for k, v in best_xgb_params.items() if k != "n_estimators"}},
        dtrain_fold,
        num_boost_round=best_xgb_params["n_estimators"],
        verbose_eval=False,
    )
    rs_xgb = xgb_risk(xgb_model, X_te)

    ci_xgb  = concordance_index_censored(ys_te["event"], ys_te["time"], rs_xgb)[0]
    auc_xgb = roc_auc_score(yb_te, rs_xgb)
    ap_xgb  = average_precision_score(yb_te, rs_xgb)
    results["XGBoost"]["cindex"].append(ci_xgb)
    results["XGBoost"]["auroc"].append(auc_xgb)
    results["XGBoost"]["auprc"].append(ap_xgb)
    fold_preds["XGBoost"]["scores"].extend(rs_xgb.tolist())
    fold_preds["XGBoost"]["labels"].extend(yb_te.tolist())
    fold_preds["XGBoost"]["times"].extend(ys_te["time"].tolist())


best_alpha_full, _, alpha_scores_full = inner_cv_alpha(
    X, y_surv, alphas, L1_RATIO, INNER_FOLDS, RANDOM_STATE
)
final_cox = None
for alpha_try in [best_alpha_full] + list(alphas[alphas > best_alpha_full][:10]):
    try:
        final_cox = CoxnetSurvivalAnalysis(
            l1_ratio=L1_RATIO, alphas=[alpha_try],
            max_iter=1000, fit_baseline_model=True
        )
        final_cox.fit(X, y_surv)
        best_alpha_full = alpha_try
        break
    except ArithmeticError:
        continue
if final_cox is None:
    raise RuntimeError("Cox failed to converge on full data.")
final_coefs = final_cox.coef_.flatten()

final_rsf = RandomSurvivalForest(random_state=RANDOM_STATE, n_jobs=-1, **best_rsf_params)
final_rsf.fit(X, y_surv)

if best_xgb_obj["objective"] == "survival:cox":
    dtrain_full = xgb.DMatrix(
        X, label=np.where(y_surv["event"], y_surv["time"], -y_surv["time"])
    )
else:
    lower_full = y_surv["time"].astype(float)
    upper_full = np.where(y_surv["event"], y_surv["time"], np.inf)
    dtrain_full = xgb.DMatrix(X)
    dtrain_full.set_float_info("label_lower_bound", lower_full)
    dtrain_full.set_float_info("label_upper_bound", upper_full)

final_xgb = xgb.train(
    {"verbosity": 0,
     **{k: v for k, v in best_xgb_obj.items()},
     **{k: v for k, v in best_xgb_params.items() if k != "n_estimators"}},
    dtrain_full,
    num_boost_round=best_xgb_params["n_estimators"],
    verbose_eval=False,
)


brier_scores = {}
for name in ("Cox", "RSF"):
    store = oof_ibs[name]
    if not store["surv_fns"]:
        brier_scores[name] = np.nan
        continue
    try:
        y_test_pooled  = np.concatenate(store["y_test"])
        te_event_times = y_test_pooled["time"][y_test_pooled["event"]]
        valid_times    = IBS_TIMES[
            (IBS_TIMES > te_event_times.min()) &
            (IBS_TIMES < te_event_times.max())
        ]
        if len(valid_times) < 2:
            raise ValueError("Too few valid IBS times")
        ibs_val = ibs_from_oof(y_surv, store["surv_fns"], y_test_pooled, valid_times)
        brier_scores[name] = ibs_val
    except Exception as e:
        brier_scores[name] = np.nan

brier_scores["XGBoost"] = np.nan


boot_all = {m: {"cindex": [], "auroc": [], "auprc": []} for m in MODEL_META}

for name in MODEL_META:
    scores_oof = np.array(fold_preds[name]["scores"])
    labels_oof = np.array(fold_preds[name]["labels"])
    times_oof  = np.array(fold_preds[name]["times"])
    valid      = ~np.isnan(scores_oof)
    scores_oof, labels_oof, times_oof = (
        scores_oof[valid], labels_oof[valid], times_oof[valid]
    )
    rng_m = np.random.default_rng(RANDOM_STATE)
    for _ in range(N_BOOTSTRAP):
        idx = rng_m.integers(0, len(scores_oof), len(scores_oof))
        sb, lb, tb = scores_oof[idx], labels_oof[idx], times_oof[idx]
        if lb.sum() == 0 or lb.sum() == len(lb):
            continue
        try:
            surv_b = make_surv_array(lb.astype(bool), tb)
            ci  = concordance_index_censored(surv_b["event"], surv_b["time"], sb)[0]
            au  = roc_auc_score(lb, sb)
            ap  = average_precision_score(lb, sb)
            boot_all[name]["cindex"].append(ci)
            boot_all[name]["auroc"].append(au)
            boot_all[name]["auprc"].append(ap)
        except Exception:
            continue

best_model_name = max(MODEL_META, key=lambda m: np.mean(boot_all[m]["cindex"]))

X_orig        = df[FEATURES].values
feature_names = FEATURES

if best_model_name == "Cox":
    shap_vals_arr = X * final_coefs

elif best_model_name == "RSF":
    def rsf_predict_fn(X_input):
        fns = final_rsf.predict_survival_function(X_input, return_array=False)
        return np.array([-fn.y.mean() for fn in fns])

    shap_vals_arr = None
    try:
        explainer_tree = shap.Explainer(rsf_predict_fn, X, algorithm="permutation")
        sv = explainer_tree(X)
        shap_vals_arr = sv.values
        if shap_vals_arr.ndim == 3:
            shap_vals_arr = shap_vals_arr[:, :, 0]
    except Exception:
        pass

    if shap_vals_arr is None:
        try:
            explainer_tree2 = shap.TreeExplainer(
                final_rsf, feature_perturbation="tree_path_dependent"
            )
            sv = explainer_tree2.shap_values(X)
            shap_vals_arr = np.array(sv).mean(axis=0) if isinstance(sv, list) else sv
        except Exception:
            pass

    if shap_vals_arr is None:
        rng_bg     = np.random.default_rng(RANDOM_STATE)
        bg_idx     = rng_bg.choice(len(X), size=min(50, len(X)), replace=False)
        background = X[bg_idx]
        explainer_kernel = shap.KernelExplainer(rsf_predict_fn, background)
        shap_vals_arr    = explainer_kernel.shap_values(X, nsamples=300, silent=True)

else:
    explainer     = shap.TreeExplainer(final_xgb)
    shap_vals_arr = explainer.shap_values(X)

mean_abs_shap = np.abs(shap_vals_arr).mean(axis=0)
top_idx      = np.argsort(mean_abs_shap)[::-1][:TOP_N_SHAP]
top_features = [feature_names[i] for i in top_idx]
top_shap     = shap_vals_arr[:, top_idx]
top_X_orig   = X_orig[:, top_idx]
short_names  = [f[:42] + "..." if len(f) > 45 else f for f in top_features]

from matplotlib.colors import LinearSegmentedColormap
shap_cmap = LinearSegmentedColormap.from_list(
    "shap", ["#1E88E5", "#7B2D8B", "#ff0051"], N=100
)
```

Supplementary Material 3. SLU Transplant Rx research Group

Rithvik Akula

Taylor Coffman

Gavin Christy

Vanisa Ezukuse

Katherine Desanti

Jatin Dhamrait

Ava DeLonais-Dick

Jake Dredge

Andrew Dugaesescu

Claire Elah Doua

Kelly Fan

Sahita Gandra

Meghana Gudapati

Sophia Heur

Spencer Hobbs

Dinesh Jaliparthi

Madeline Johnson

Michael Kanan

Leo Lai

Cayden Lawrence

Yuan Liu

Yanqing Lyu

Anata Manjula Hasini Sriram

Khang Nguyen

Nathaniel Nguyen

Barbara Okeke

William Park

Zalan Shah

Zaman Shah

Eric Stein

Sathvik Suryadevara

Elsa Treiber

Sneha Manikandan

Reem Farid

Lanerica Rogers

Abdelrahman Gadallah

Supplementary Figure 1. Multiple imputation diagnostics: convergence trace plots and observed-versus-imputed density overlays for variables with missing data
Abbreviations: BMI = body mass index; eGFR = estimated glomerular filtration rate; LA = left atrial; LV = left ventricular; LVEF = left ventricular ejection fraction; MELD = Model for End-Stage Liver Disease; MICE = multivariate imputation by chained equations; RIMP = right ventricular index of myocardial performance; RV = right ventricle; RVFAC = right ventricular fractional area change; TR = tricuspid regurgitation

Supplementary Figure 2. Competing-risk analysis of post-transplant major adverse cardiovascular events
Abbreviations: CI = confidence interval; CIF = cumulative incidence function; CR = competing risk; IPCW = inverse probability of censoring weighting; KM = Kaplan-Meier; MACE = major adverse cardiovascular event; non-CV = non-cardiovascular; RSF = random survival forest

Supplementary Figure 3. Per-variable missingness rates across included model features
Abbreviations: BMI = body mass index; eGFR = estimated glomerular filtration rate; LA = left atrial; LV = left ventricular; LVEF = left ventricular ejection fraction; MCAR = missing completely at random; MELD = Model for End-Stage Liver Disease; RIMP = right ventricular index of myocardial performance; RV = right ventricle; RVFAC = right ventricular fractional area change; TR = tricuspid regurgitation

Supplementary Figure 4. Elastic net Cox regularization path

Supplementary Figure 5. Kaplan-Meier event-free survival, bootstrap cluster stability, and major adverse cardiovascular event rate by multidomain phenotype
Abbreviations: ARI = adjusted Rand index; CI = confidence interval; MACE = major adverse cardiovascular event

Supplementary Figure 6. Strain-only echocardiographic subphenotypes derived from left ventricular global longitudinal strain, left atrial reservoir strain, and right ventricular free wall strain
Abbreviations: ARI = adjusted Rand index; LASr = left atrial reservoir strain; LV GLS = left ventricular global longitudinal strain; MACE = major adverse cardiovascular event; RVSfw = right ventricular free wall strain

Supplementary Figure 7. Phenotype-augmented model performance
Abbreviations: AP = average precision; AUC = area under the receiver operating characteristic curve
